# Collapsed polymer-directed synthesis of multicomponent coaxial-like nanostructures

**DOI:** 10.1038/ncomms12147

**Published:** 2016-07-19

**Authors:** Zhiqi Huang, Yijing Liu, Qian Zhang, Xiaoxia Chang, Ang Li, Lin Deng, Chenglin Yi, Yang Yang, Niveen M. Khashab, Jinlong Gong, Zhihong Nie

**Affiliations:** 1 Key Laboratory for Green Chemical Technology of Ministry of Education, School of Chemical Engineering and Technology, Tianjin University Collaborative Innovation Center of Chemical Science and Engineering, Tianjin 300072, China; 2 Department of Chemistry and Biochemistry, University of Maryland College Park, Maryland 20742, USA; 3 Smart Hybrid Materials Laboratory, Advance Membranes and Porous Materials Center, King Abdullah University of Science and Technology, Thuwal 23955-6900, Kingdom of Saudi Arabia

## Abstract

Multicomponent colloidal nanostructures (MCNs) exhibit intriguing topologically dependent chemical and physical properties. However, there remain significant challenges in the synthesis of MCNs with high-order complexity. Here we show the development of a general yet scalable approach for the rational design and synthesis of MCNs with unique coaxial-like construction. The site-preferential growth in this synthesis relies on the selective protection of seed nanoparticle surfaces with locally defined domains of collapsed polymers. By using this approach, we produce a gallery of coaxial-like MCNs comprising a shaped Au core surrounded by a tubular metal or metal oxide shell. This synthesis is robust and not prone to variations in kinetic factors of the synthetic process. The essential role of collapsed polymers in achieving anisotropic growth makes our approach fundamentally distinct from others. We further demonstrate that this coaxial-like construction can lead to excellent photocatalytic performance over conventional core–shell-type MCNs.

## Abstract (web summary)

Multicomponent colloidal nanostructures have topologically dependent chemical and physical properties, but are difficult to synthesise with high order complexity. Here, Nie and co-workers show a general and scalable route to synthesise such structures with unique coaxial-like construction.

## Graphical abstract

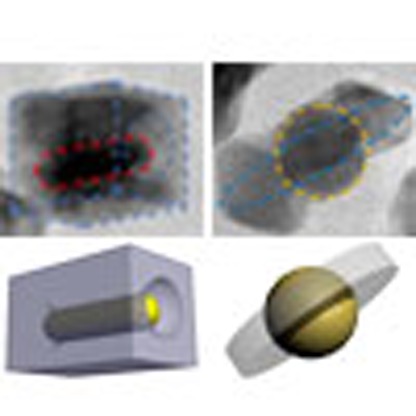

Multicomponent colloidal nanostructures (MCNs) naturally integrate two or more different materials (for example, metals, oxides and semiconductors) into one system with nanoscale domains. These MCNs inherit the intrinsic properties of each constituent component, which makes them multifunctional^1^. Furthermore, new physical and chemical properties that are different from any of the single component alone may arise from the synergetic effect of the multiple directly contacted (or closely placed) disparate components^2^,^3^. As a result, MCNs show a broad range of emerging applications in catalysis^4^, solar energy harvesting^5^, nanoelectronics^6^, biomedical diagnosis and therapy^7^. The spatial distribution of dissimilar components within individual MCNs is crucial in defining the properties and hence applications of these MCNs^8^. For instance, ternary MCNs of Au–Ag, CdSe and Pt domains with linear or triangular configuration show distinct photocatalytic performance, as the spatial arrangement of constituent components regulates the electron pathway in the nanoscale system and thus the reaction mechanism^9^. The most commonly engineered architectures of MCNs are core–shell geometry with an inner ‘core' embedded within a ‘shell' of other types of materials^10^ and oligomer-type configuration with spatially asymmetric components interconnected through bonding interfaces^11^. MCNs with coaxial-like arrangement of constituent components uniquely combine the architectural features of both core–shell and oligomer-type topologies, which may result in new properties and applications that are not accessible otherwise^12^,^13^.

The most widely exploited wet-chemical strategy for synthesizing MCNs is the so-called ‘seeded-growth' method in which a secondary inorganic material is selectively deposited onto the surface of a primary seed nanoparticle (NP)^14^. The growth mode of a secondary material and the structure of resulting MCNs can be largely deduced from the equilibrium consideration of interface energy arising from lattice mismatch between the two materials^15^. In order to generate MCNs with controlled architectures, appropriate organic molecules or ions are used to tune the energy balance, thus achieving selective local deposition^16^. Alternatively, kinetic control can also be employed to promote the anisotropic deposition^17^. These synthetic strategies have been used to synthesize compositionally and geometrically diverse MCNs, ranging from concentric/eccentric core–shell geometries to asymmetric hetero-oligomer architectures. However, the synthesis of coaxial-like nanostructures with different material combinations has been rarely reported^18^.

This paper describes a polymer-assisted seeded-growth strategy for the synthesis of coaxial-like MCNs comprising a shaped Au core surrounded by a tubular metal or metal oxide shell. Polymers locally collapsed on the surface of Au nanorods or nanospheres form immobilized rigid blocks because of their conformational change in response to the variation in solvent quality. These polymer blocks serve as effective protection layer on the Au NP seed to guide the selective deposition and growth of a secondary material on the unprotected surfaces. By using this approach, we produce a gallery of coaxial-like MCNs comprising Au nanorods or nanospheres as inner core and metal (for example, Ag, Pt, Pd and Ni) or metal oxide (for example, Cu_2_O and CeO_2_) as shielding layer. The thickness of the shell and the shape of overall coaxial-like MCNs can be controlled by varying reactant concentrations or choosing appropriate surfactants. In all these cases, the tips of the coaxial-like MCNs are deprived of inorganic deposition and the collapsed polymers on MCN surfaces can be re-solubilized in organic solvent to stabilize the MCNs (or removed). Our synthetic approach is robust and reproducible, and it is not sensitive to variations in kinetic factors (for example, reaction rate) owing to the efficient protection of collapsed polymers. The essential role of collapsed polymers in achieving anisotropic growth makes our approach fundamentally distinct from others. This general strategy can be extended for the synthesis of other highly complex anisotropic MCNs, which are otherwise difficult to make.

## Results

### Concept

Polymers are known to change their confirmation in response to the variation in solvent quality. This phenomenon has been widely used to produce complex polymer assemblies with programmable nanoscale architectures^19^,^20^. Here we introduce polymers as bi-functional ligands in the synthesis of MCNs with desired geometry. We demonstrate that, when tethered to inorganic NP seeds, linear polymers can be either stretched (that is, solubilized) to stabilize the NPs, or collapsed to serve as geometry-directing agents for the anisotropic deposition of another material by taking advantage of their ‘smart' conformational change. Compared with polymer-guided assembly of NPs in previous work^21^, the current work represents an important step forward of utilizing polymers to control the synthesis of MCNs with tailored architectures.

**Figure 1:**
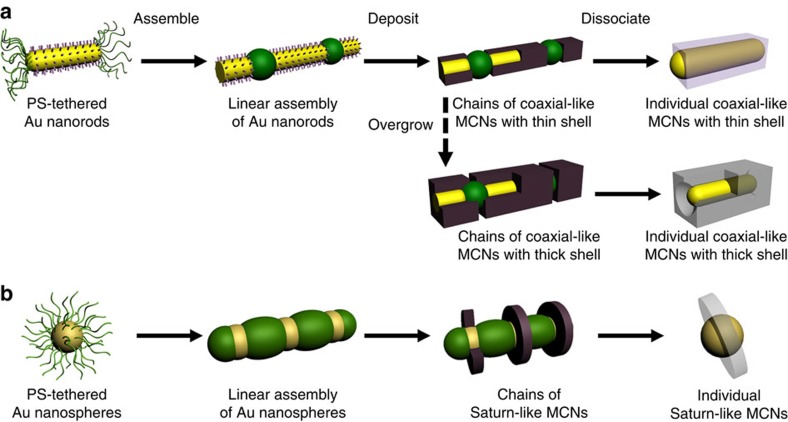
Schematic illustration of collapsed polymer-directed growth of coaxial-like MCNs. (**a**,**b**) The synthetic route when anisotropic Au nanorods (**a**) or isotropic Au nanospheres (**b**) are used as seeds for the selective metal or oxide deposition.

We use seeds of Au nanorods as a model system to illustrate the concept of our synthesis (Fig. 1a). Cetyltrimethylammonium bromide (CTAB)-covered Au nanorods are first surface-modified with thiol-terminated hydrophobic polystyrene (PS, *M*_n_=12.0 k, polydispersity index=1.09) in tetrahydrofuran (THF) through ligand exchange^21^. The PS chains are preferentially tethered to both ends of Au nanorods because of the deprivation of CTAB on those surfaces^22^,^23^, and serve as stabilizing ligands. The PS-tethered Au nanorods in dimethylformamide (DMF) are assembled into one-dimensional chains upon the introduction of water—a poor solvent for PS because of the association of hydrophobic PS on neighbouring NPs to minimize interfacial energy^21^. The associated PS locally collapses between adjacent NPs and forms rigid blocks covering the ends of Au nanorods. The Au nanorod chains are subject to secondary growth in an aqueous solution of metal or metal oxide precursors. The collapsed PS blocks serve as a rigid protective layer to efficiently isolate the ends from surrounding media, while the CTAB-covered sides of the seeds are exposed. The inaccessibility of growth media to protected areas leads to the selective deposition of metal or metal oxides only on the sides of the seeds. After the growth, the chains of MCNs can be dissociated as isolated coaxial-like MCNs by re-solubilizing the PS blocks—which serve as stabilizing ligand again—with organic solvents such as THF and DMF. The same principle can be applied to the synthesis of MCNs with Saturn-like construction by using spherical Au NPs as seeds (Fig. 1b).

### Coaxial growth of Pd on Au nanorods

**Figure 2:**
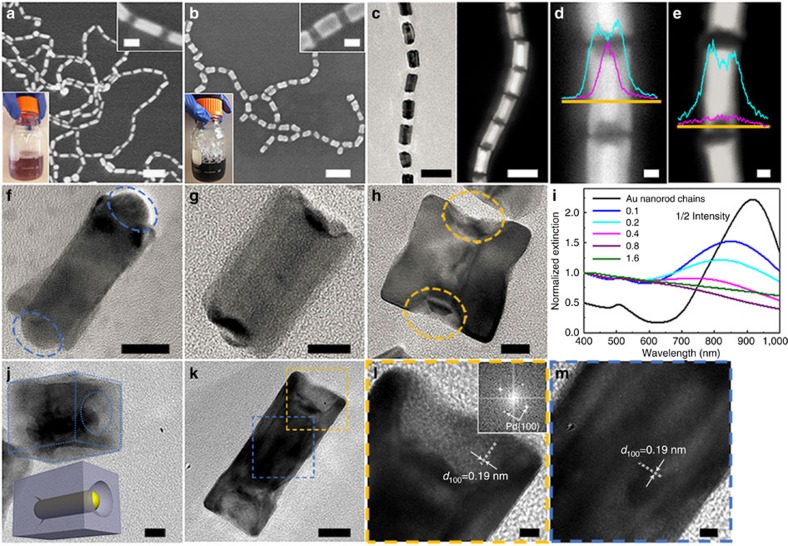
Coaxial-like MCNs with Au nanorod cores and tubular Pd shells. (**a**,**b**) SEM images of Au nanorod chains before (**a**) and after (**b**) the selective deposition of Pd. Top-right insets are corresponding zoom-in SEM images, while bottom-left insets are optical images of the product solutions. (**c**) TEM and HAADF STEM images of chains of coaxial-like MCNs. (**d**,**e**) EDS line scan of an individual coaxial-like MCN at different regions showing the distribution of Au (purple) and Pd (cyan). (**f**–**h**) HRTEM images of individual coaxial-like MCN with increasing shell thicknesses, where blue circles highlight the exposed ends of Au nanorods and yellow circles highlight the cavities at the tips of MCNs. (**i**) UV–vis extinction spectrum of chains of coaxial-like MCNs with different shell thicknesses. The MCNs were synthesized using 0.1, 0.2, 0.4, 0.8 and 1.6 ml of H_2_PdCl_4_ solution. (**j**) HRTEM image of a tilted individual coaxial-like MCN with thick Pd shell. Dashed blue lines outline the cuboid morphology and cavity at the tip. Inset is the corresponding structural model. (**k**) HRTEM image of a single coaxial MCN. (**l**,**m**) Zoom-in HRTEM images of the yellow and blue squares in **k** showing the Pd (100) lattice planes at the tip and in the middle of a MCN, respectively. Inset in **l** is the corresponding Fast Fourier Transform image showing a typical orthogonal Pd (100) pattern. Scale bars, 100 nm in **a**,**b**, 20 nm in insets of **a**,**b**, 50 nm in **c**,**d**, 10 nm in **d**–**k** and 2 nm in **l**,**m**.

Palladium (Pd)-coating is chosen as a model to demonstrate the concept. Figure 2a,b shows typical scanning electron microscope (SEM) images of Au nanorod chains before and after they were subjected to metal deposition in an aqueous solution containing Pd precursors and CTAB (also see Supplementary Fig. 1)^24^. The selective deposition of Pd on the sides of Au nanorods was evidenced by an obvious colour change of the solution from reddish brown to dark black (bottom-left insets of Fig. 2a,b) and a significant increase in the rod diameter (top-right insets of Fig. 2a,b and Supplementary Fig. 2). A close inspection by regular transmission electron microscopy (TEM) and high-angle annular dark-field TEM (HAADF-TEM) indicated that Pd was coaxially coated along the longitudinal sides of Au nanorods to form coaxial-like MCNs with a tubular Pd shell while leaving both ends exposed (Fig. 2c and Supplementary Fig. 2). The distribution of Au and Pd along the transverse direction of a coaxial-like MCN was further characterized by energy-dispersive X-ray spectrometer (EDS) line scan at different positions of a single coaxial-like MCN (Fig. 2d,e). In the middle of a MCN, the sandwich of an Au peak between two Pd peaks suggests the growth of Pd on the sides of Au nanorods, whereas, at the tip, the presence of only two Pd peaks indicates the formation of Pd tubular structures without Au core, that is, both ends of the Au nanorod core of MCNs are deprived of Pd deposition. The coaxial growth of Pd can be explained by the protection of both ends of Au nanorods with collapsed polymer blocks and the unfavourable nucleation of Pd on polymer domains because of the large interfacial energy between Pd and polymer. Isolated individual MCNs could be redispersed in good solvent (for example, DMF and THF) for the hydrophobic PS by dissociating the hybrid chains (Supplementary Fig. 3). This approach could be readily scaled up while maintaining same quality of MCNs (bottom-left insets of Fig. 2a,b).

The thickness of Pd shell can be precisely tuned by controlling the amount of Pd precursor (Supplementary Fig. 4). The diameter of MCNs approximately increased from 14 to 35 nm linearly with the square root of Pd precursor amount (Supplementary Figs 5 and 6). This scaling law further confirms the formation of tubular Pd shell on Au nanorods (see Supplementary Methods for detailed estimation). When a thin layer of Pd was deposited on Au nanorods, both ends of Au nanorods were extruded from the Pd shell and completely exposed (Fig. 2f). Further deposition of Pd on the MCNs led to the propagation of Pd towards polymer domains (Fig. 2g) and eventually to the formation of cavities that were filled with collapsed polymers at both ends of nanorods (Fig. 2h and Supplementary Figs 6 and 7). As a result of the increase in the Pd shell thickness and decrease in the aspect ratio of the overall MCNs, the localized surface plasmon resonance (LSPR) of the MCNs gradually blue-shifted and suppressed, and eventually disappeared (Fig. 2i). High-resolution TEM (HRTEM) image of a tilted coaxial-like MCN with thick Pd shell in Fig. 2j highlights the cavities at the tips and reveals a cuboid feature of the tubular shell (also see Supplementary Fig. 8). The tubular cuboid Pd shell is primarily enclosed by (100) facet and is monocrystalline throughout the entire shell when CTAB is used as surfactant (Fig. 2k)^24^. The lattice planes both at the tip (Fig. 2l) and in the middle (Fig. 2m) of a MCN could be assigned to the Pd (100) facet. This is also confirmed by Fast Fourier Transform analysis (inset of Fig. 2l).

The shell morphologies can be readily controlled by choosing appropriate precursors and surfactants^25^,^26^. The presence of extra amount of HCl in the growth solution led to the formation of truncated octahedron shell (Supplementary Fig. 9), while coaxial-like MCNs with jagged shell were produced when Na_2_PdCl_4_ instead of H_2_PdCl_4_ was used as the Pd precursor (Supplementary Fig. 10). It is known that HCl can promote the oxidative etching of noble metals^27^. We, therefore, ascribe the formation of jagged shell or truncated shell to the relative rate of etching and regrowth of Pd. When CTAB was replaced with cetyltrimethylammonium chloride as surfactant, the deposition produced MCNs with porous Pd shell (Supplementary Figs 11–13). This can be explained by a fast reduction rate of Pd as a result of the higher redox potential of [PdCl_4_]^2−^/Pd^0^ pair (0.62 eV) than that of [PdBr_4_]^2−^/Pd^0^ pair (0.49 eV; ref. 28). Notably, in spite of different reaction kinetics in these cases, the ends of Au nanorod cores were all well protected in the course of metal deposition. This highlights the robustness of our approach in directing anisotropic growth of MCNs.

We note that the use of collapsed polymers is crucial to the formation of coaxial-like MCNs. Au nanorods coated with hydrophilic thiol-terminated poly (ethylene oxide) of similar polymer chain length failed in producing coaxial MCNs with exposed ends of Au nanorods, regardless of the graft density of polymers on the rod seeds and the amount of Pd precursor (Supplementary Figs 14 and 15). Instead, nucleation and growth of Pd occurred all over Au nanorods to generate core–shell Au@Pd nanorods, indicating that swelling hydrophilic poly (ethylene oxide) chains were insufficient to block the access of precursors to Au surface in an aqueous system^29^.

### Gallery of coaxial-like MCNs with various compositions

**Figure 3:**
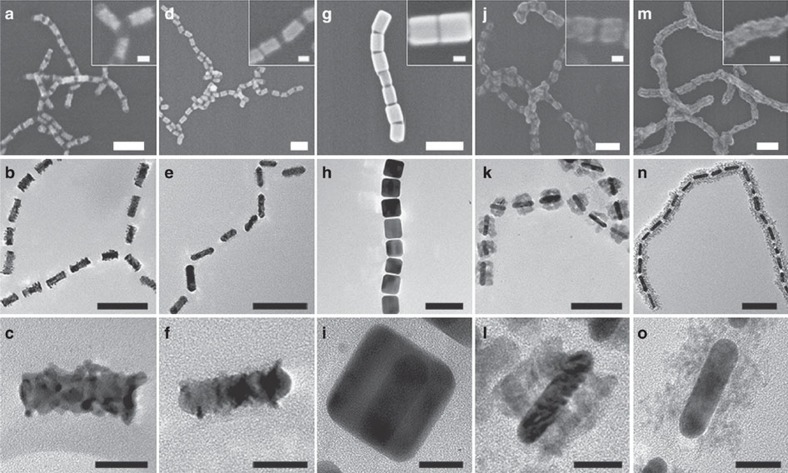
Gallery of coaxial MCNs with Au nanorod cores and various metal or oxide shells. (**a**–**o**) SEM, TEM and HRTEM images of coaxial MCNs with different shell materials: Pt (**a**–**c**), Pt/Ni (**d**–**f**), Ag (**g**–**i**), Cu_2_O (**j**–**l**) and CeO_2_ (**m**–**o**). Insets are corresponding high-resolution SEM (HRSEM) images. Scale bars, 100 nm in SEM and TEM images and 20 nm in HRSEM and HRTEM images.

To demonstrate the generality of our strategy, various inorganic materials were used to deposit on Au nanorod chains (Fig. 3). Noble metal such as platinum (Pt) and silver (Ag), base metal nickel (Ni), transition metal oxides such as cuprous oxide (Cu_2_O) and cerium oxide (CeO_2_) were successfully coated on the sides of Au nanorods to form coaxial-like structures. The deposition of Pt led to the formation of multicrystalline dendrite-like tubular shell on the side of Au nanorods (Fig. 3a–c and Supplementary Fig. 16) because of the large lattice mismatch between Pt and Au and the self-catalysed growth nature of Pt (ref. 30). Similarly, coaxial-like Au–Pt/Ni MCNs also exhibited dendrite-like feature (Fig. 3g–i). The Pt-catalysed Ni deposition was confirmed by quantitative analysis of shell thickness (Supplementary Figs 17–19) and TEM elemental analysis (Supplementary Fig. 20). The UV–vis extinction spectrum of these MCNs showed similar blue-shift and suppression of LSPR peaks with the increase in shell thickness (Supplementary Figs 21 and 22), as in the case of Pd growth. In contrast, the deposition of Ag led to the formation of uniform and smooth Ag shell, as a result of epitaxial growth of Ag on Au (Fig. 3g–i and Supplementary Fig. 23)^31^. The deposition process was accompanied with the initial blue-shift and then red-shift of the LSPR peak. This can be explained by the competitive effects: increase in the rod diameter and decrease in the end-to-end distance between adjacent MCNs (Supplementary Fig. 24)^22^.

The same method was used to guide the selective condensation of oxides on the sides of Au nanorods to produce coaxial-like MCNs with dense Cu_2_O shell and porous CeO_2_ shell. In the case of Cu_2_O, the relative small lattice mismatch between Au and Cu_2_O (4.5%) allows the epitaxial growth of Cu_2_O on Au (ref. 32). A cubic Cu_2_O phase was confirmed by HRTEM images (Fig. 3j–l and Supplementary Fig. 25). The CeO_2_ shell was also formed coaxially along the longitudinal direction of the chains. However, the shell was porous because of the large mismatch between Au and CeO_2_ (ref. 33) and preferred to cover the whole chain, especially when the shell was thick (Fig. 3m–o and Supplementary Fig. 26). Nevertheless, the ends of Au nanorods were well protected from oxide condensation in both cases. The coating of oxides led to a red-shift of LSPR, as a result of the large refractive index of oxides^34^ (Supplementary Figs 25c and 27). All above-mentioned chains of coaxial-like MCNs could be readily dissociated into isolated individual coaxial-like MCNs in organic solvents (Supplementary Fig. 28). The coaxial-like MCNs were stable for more than 1 week either in the form of chains or as individual MCNs, as revealed by dynamic light scattering, UV–vis analysis and TEM imaging analysis (Supplementary Fig. 29).

### Coaxial growth on Au nanospheres

**Figure 4:**
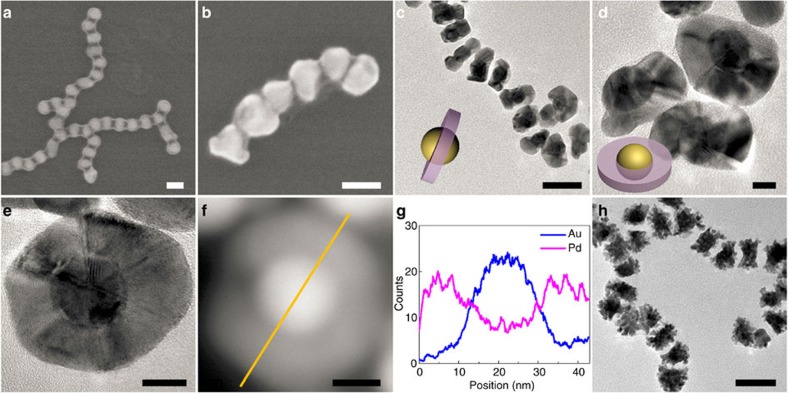
Saturn-like MCNs with Au nanospheres as cores and metal shells. (**a**) SEM image of original Au nanosphere chains. (**b**,**c**) SEM (**b**) and TEM (**c**) images of chains of Au–Pd Saturn-like MCNs with an Au nanosphere core and a Pd tubular shell. (**d**,**e**) TEM (**d**) and HRTEM (**e**) images of dissociated Au–Pd Saturn-like MCNs. (**f**) HAADF STEM images of an individual MCN and (**g**) EDS line scan along the line in **f**. (**h**) TEM image of chains of Au–Pt Saturn-like MCNs. Scale bars, 50 nm in **a**–**c**; 10 nm in **d**–**f**; and 50 nm in **h**.

Our approach is applicable to the coaxial growth of metal on spherical Au NPs with isotropic surface. CTAB-capped Au nanospheres grafted with low density of PS were assembled into chains with PS collapsed between neighbouring NPs along the chain, while leaving the side surface of Au nanospheres unprotected (Fig. 1b and Fig. 4a). The formation of nanosphere chains can be largely ascribed to the competitive effect of electrostatic repulsion and hydrophobic attraction between neighbouring Au nanospheres coated with both hydrophobic PS and charged CTAB ligands^35^. The anisotropic phase separation of polymer domains on Au nanosphere surfaces enables the selective deposition of a second inorganic material onto isotropic Au nanospheres. Taking Pd growth as an example, the coaxial deposition on the unprotected sides produced Saturn-like nanostructures with Au nanosphere core and Pd tubular shell. As shown in Fig. 4b, the diameter of Au nanosphere chains increased from 20 to ∼40 nm after the deposition of Pd (see SEM/TEM images and corresponding UV–vis measurements in Supplementary Figs 30 and 31). Side and top-down views of individual coaxial-like MCN reveal a disk-like feature and a Saturn-like configuration of the nanostructures (Fig. 4c,d and Supplementary Fig. 32). HRTEM and HAADF-TEM images of a single Saturn-like MCN clearly show distinct Au and Pd domains where the Au domain is surrounded by the Pd domain (Fig. 4e,f). The formation of coaxial-like architecture of MCNs was further confirmed by the sandwiched peaks in EDS line scan in Fig. 4g. To the best of our knowledge, this is the first demonstration of the fabrication of MCNs with Saturn-like construction. Other than Pd, other metals such as Pt and Ag were also used as shell material to produce MCNs with similar Saturn-like geometry (Fig. 4h and Supplementary Figs 33–35).

### Photocatalytic performances of coaxial-like MCNs

**Figure 5:**
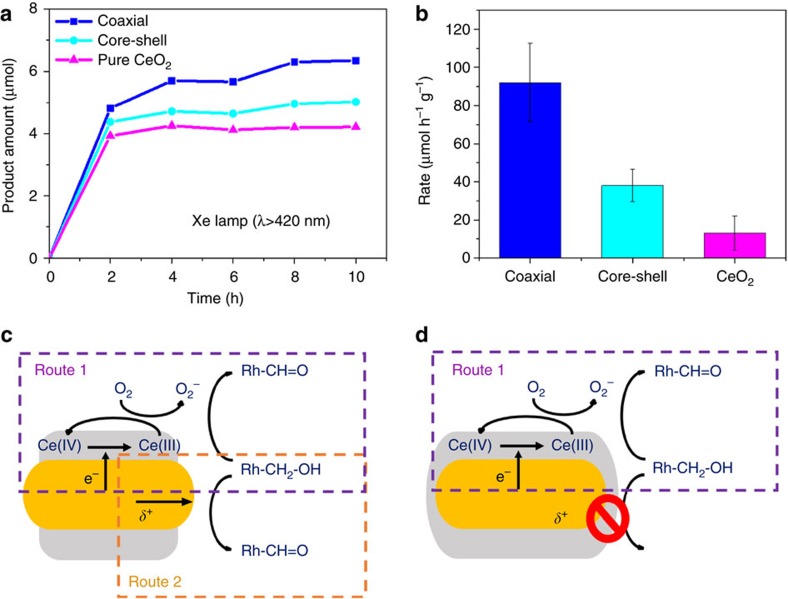
Photo-oxidation of benzyl alcohol to benzaldehyde with coaxial-like and core–shell-type Au–CeO_2_ MCNs as catalysts. (**a**) The amount of benzaldehyde as a function of time by using coaxial-like, core–shell-type Au–CeO_2_ MCNs and pure CeO_2_ NPs as catalysts. (**b**) Calculated rate of benzaldehyde generation for the last 8 h. The results were normalized to the mass of catalysts. (**c**,**d**) Proposed photo-oxidation mechanisms for coaxial-like Au–CeO_2_ MCNs (**c**) and core–shell-type Au–CeO_2_ MCNs (**d**), respectively. The dramatic high activity in the first two hours of all catalysts can be attributed to the contribution of intrinsic Ce(III) species in CeO_2_. Pure CeO_2_ became inactive thereafter because of the depletion of Ce(III) species.

Rational design of Au nanorod-based MCNs has shown great potential in promoting the spatial separation of photogenerated opposite charge carriers, thus achieving enhanced photocatalytic performance^12^,^36^. Here we compared the performance of Au–CeO_2_ MCNs with coaxial-like and core–shell constructions in catalysing the selective photo-oxidation of benzyl alcohol to benzaldehyde. The reaction was conducted at ambient condition with visible light irradiation and cooling water to eliminate the thermal effect (see Methods for experimental details). As shown in Fig. 5a, MCNs with the coaxial-like construction showed more efficient generation of benzaldehyde compared with those with core–shell geometry. Ignoring the contribution of intrinsic Ce(III) species in CeO_2_ in the first two hours^33^, the rate of benzaldehyde generation by the coaxial-like MCNs was over two times higher than that by the core–shell MCNs (Fig. 5b). We attribute the enhanced photocatalytic activity of our coaxial-like MCNs to the efficient utilization of photogenerated charge carriers (hot electrons and holes) owing to the exposure of both CeO_2_ shells and Au cores to the solution media. As illustrated in Fig. 5c, the coaxial-like construction allows two reaction routes towards benzaldehyde generation: (1) photogenerated hot electrons are injected from Au core to CeO_2_, reducing Ce(IV) to Ce(III). The Ce(III) species react with dissolved oxygen and generate superoxide, which thereafter oxidize benzyl alcohol to benzaldehyde; (2) positive charges accumulated on Au cores can directly lead the alcohol oxidation reaction on the exposed Au nanorod tips^37^,^38^. Thus, both charge carriers contribute to the benzaldehyde generation. Whereas in the case of Au–CeO_2_ MCNs with conventional core–shell construction (Supplementary Fig. 36), the full CeO_2_ coverage prevents the access of reactants to Au surfaces and hence reacting with positive charges, that is, only Route 1 is available for the catalytic reaction (Fig. 5d).

The advantage of our coaxial-like construction was further demonstrated in the photodegradation of methylene blue using bimetallic Au–Pt MCNs. The coaxial-like Au–Pt MCNs exhibited 1.6 times higher first-order kinetic rate constant, compared with core–shell-type Au@Pt MCNs (Supplementary Fig. 37). We presume that this is because of the continuous scavenging of accumulated positive charges through exposed Au core, which can efficiently reduce the recombination of hot electrons and holes^9^,^39^.

## Discussion

In conclusion, we have developed an efficient and robust approach for the synthesis of complex MCNs using bi-functional polymers as geometry-directing agents and stabilizing ligands, by taking advantage of their smart conformational change. We successfully synthesized diverse Au–metal (or metal oxide) MCNs with unique coaxial-like (or Saturn-like) construction that is not readily attainable otherwise. The size, shape, composition and morphology of both the core and coaxial shell can be precisely controlled. Other metals such as Pd and Ag with more elaborate shapes^40^,^41^ can be potentially used as seeds to generate even more complicated hybrid nanostructures, in combination with other techniques, such as cation exchange^42^, Kirkendall effect^43^ and oxidative etching^44^. This unique capability of controlling nanoscale geometry of MCNs offers a platform to study structure-dependent synergetic properties of MCNs. As a demonstration, coaxial-like MCNs were used as photocatalysts and showed superior performance compared with conventional core–shell-type MCNs. Moreover, these MCNs with topologically complex features may find a broad range of applications in such as solar energy harvesting^12^, catalysis^45^ and biomedical applications^46^.

## Methods

### Preparation of Au nanorod chains

Au nanorods were prepared according to previous literature with slight modification^47^. The detailed preparation procedure is given in Supplementary Methods. To prepare Au nanorod chains, a 50-ml solution of as-synthesized Au nanorods was first concentrated to 0.3 ml by centrifugation. The solution was added dropwise into a 10-ml solution of thiol-terminated PS in THF with a concentration of 1 mg ml^−1^. The solution was sonicated for half an hour and incubated overnight at room temperature. After six cycles of washing with THF, the PS-modified Au nanorods were redispersed in 5 ml DMF in a 20-ml vial. A 5-ml solution of DMF/water (60% wt/40% wt) was then added dropwise under gentle shaking, and the solution was incubated overnight. The mixture solution was dialysed against 800 ml of 50 mM CTAB aqueous solution using dialysis membrane tubing (molecular-weight cutoff of 6,000–8,000 g mol^−1^) for 2 h to further stabilize the chains, followed by centrifugation at 7,000 r.p.m. for 15 min. The precipitates were redispersed in 10 ml of water. The aqueous solution of Au nanorod chains was used for all secondary growth.

### Synthesis of coaxial-like Au–Pd MCNs with cuboid shell

A 10-mM H_2_PdCl_4_ solution was prepared by dissolving 0.036 g of PdCl_2_ powder in 10 ml of 50 mM HCl solution and diluting to 20 ml. For cuboid Pd shell growth with different shell thicknesses, a 1-ml aqueous solution of Au nanorod chains was mixed with 1 ml of 0.2 M CTAB in a 20-ml vial. Then, appropriate amount of water, H_2_PdCl_4_ and ascorbic acid solution were sequentially added to make the final volume 10 ml. After gentle shaking, the vial was capped and left in a 30-°C water bath for 10 h. The samples after growth were washed twice with water before characterization and further use. For scaled up synthesis, all solutions were amplified by 10 times, and the synthesis was carried out in a 250-ml glass bottle.

### Photocatalytic oxidation of benzyl alcohol to benzaldehyde

As-synthesized coaxial-like Au–CeO_2_ MCNs were washed twice with water and twice with DMF, and then dissolved in THF. The THF solution was sonicated for 1 h at 70 °C and washed with THF. The sonication and washing process was repeated for three times to remove the polymers. All catalysts were dried in air at 90 °C for 5 h. Then, 2.0 mg of photocatalysts (coaxial-like, core–shell-type Au–CeO_2_ MCNs and pure CeO_2_ NPs) were added in a home-made reactor sealed with rubber stoppers. Then, 5 ml of toluene (solvent), 50 μl of benzyl alcohol (reactant) and 40 μl of undecane (internal standard) were injected into the reactor. O_2_ was bubbled through the mixture at 20 ml min^−1^. The reactor was irradiated under magnetic stirring using a 300-W xenon lamp (*λ*>420 nm) with an irradiation area of 6.25 cm^2^ and an irradiation intensity of 60 mW cm^−2^. The reaction was then conducted at room temperature with cooling water used to eliminate the thermal effect.

### Characterizations

UV–vis extinction measurements were performed using the PERKIN LAMBDA 40 UV–vis system. The structure and morphology of MCNs were analysed with Hitachi SU-70 Analytical Field Emission Gun SEM operated at 15 kV and JEM 2100 LaB6 TEM operated at 200 kV accelerating voltage. Single-point EDS measurements were operated under scanning transmission electron microscopy mode using the JEOL 2100F. HAADF images and EDS line scan were performed on JEOL 2100F TEM using the scanning transmission electron microscopy mode. All the SEM samples were prepared by dispensing a drop of the MCN solution on silicon wafers and drying at room temperature. All the TEM samples were prepared by depositing a drop of MCN solution on 400 mesh carbon-coated copper grids and allowing solvent evaporation at room temperature. The photocatalytic products were analysed with a gas chromatograph system (GC 2060, Ramiin) with a flame ionization detector. A standard solution (a mixture containing 5 ml of toluene, 50 μl of benzyl alcohol, 40 μl of benzaldehyde and 40 μl of undecane) was used to calibrate the GC. The calibration factor (1.7) was obtained using the standard solution. The accurate amount of every sample was calculated using the area of every peak and the calibration factor. The correlation of peaks and products is determined by the retention time obtained from the prior analysis of pure chemicals.

### Data availability

The authors declare that the data supporting the findings of this study are available within the article and its Supplementary Information files.

## Additional information

**How to cite this article**: Huang, Z. *et al*. Collapsed polymer-directed synthesis of multicomponent coaxial-like nanostructures. *Nat. Commun.* 7:12147 doi: 10.1038/ncomms12147 (2016).

## Supplementary Material

Supplementary Information Supplementary Figures 1-37, Supplementary Methods and Supplementary References
