# Supplementary material for: Collapsed polymer-directed synthesis of multicomponent coaxial-like nanostructures
Source: Nat Commun. 2016 Jul 19;7:12147. doi: 10.1038/ncomms12147 (PMC4960297; doi:10.1038/ncomms12147)
Supplement: Supplementary Information — Supplementary Figures 1-37, Supplementary Methods and Supplementary References [file ncomms12147-s1.pdf]

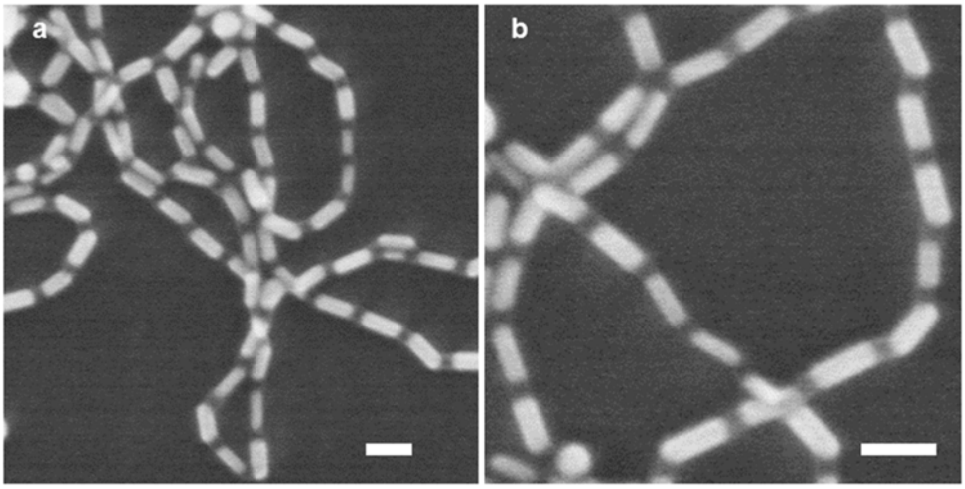

Supplementary Figure 1 | SEM images of Au nanorod chains with distinct Au nanorods and polymer domains. The Au nanorods are associated in an end-to-end manner to form linear chains with collapsed polymer domains located between neighboring nanorods. Scale bars are 50 nm.

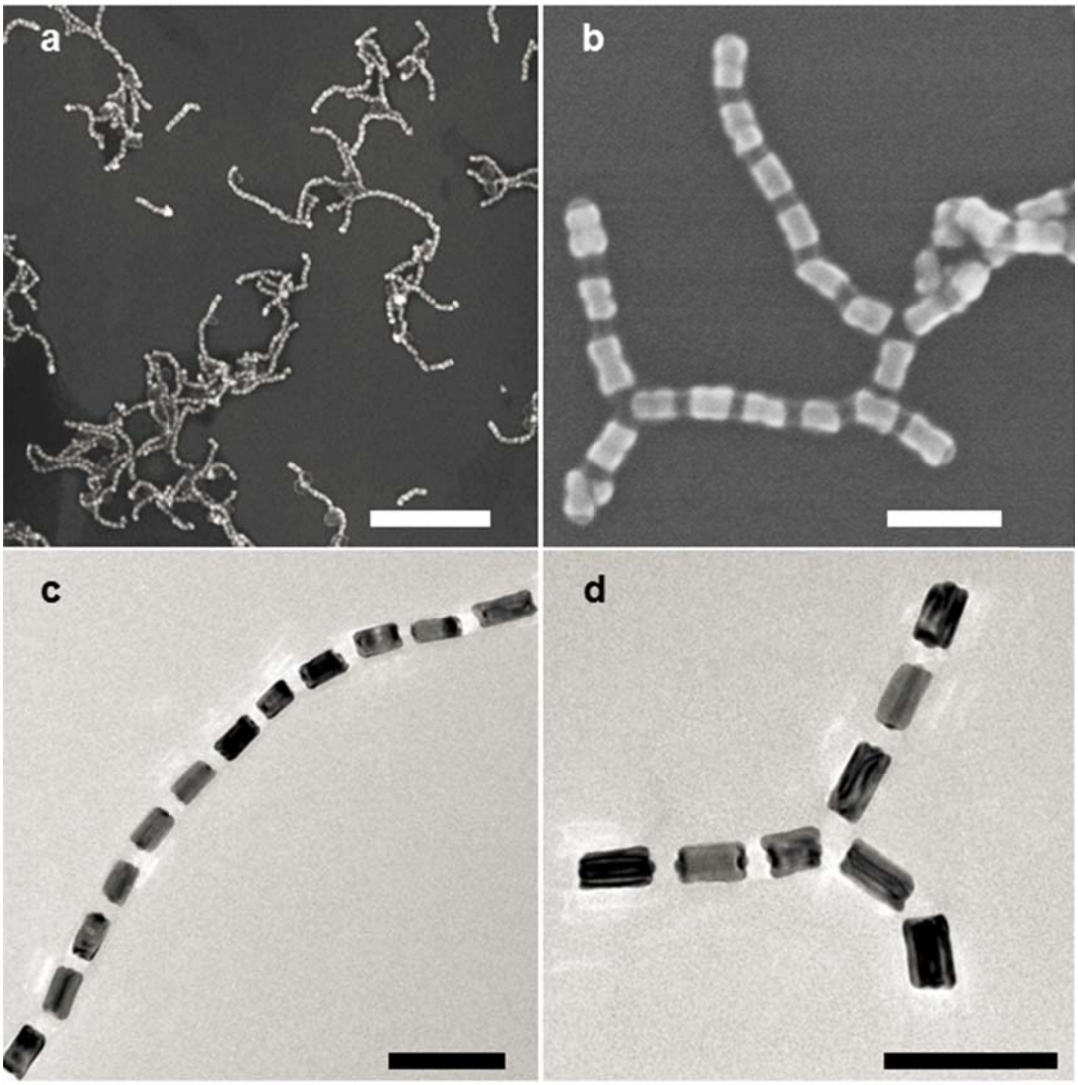

Supplementary Figure 2 | SEM and TEM images of chains of coaxial-like Au-Pd MCNs.

Scale bars are 1 μm in **a**, and 100 nm in **b**-**d**.

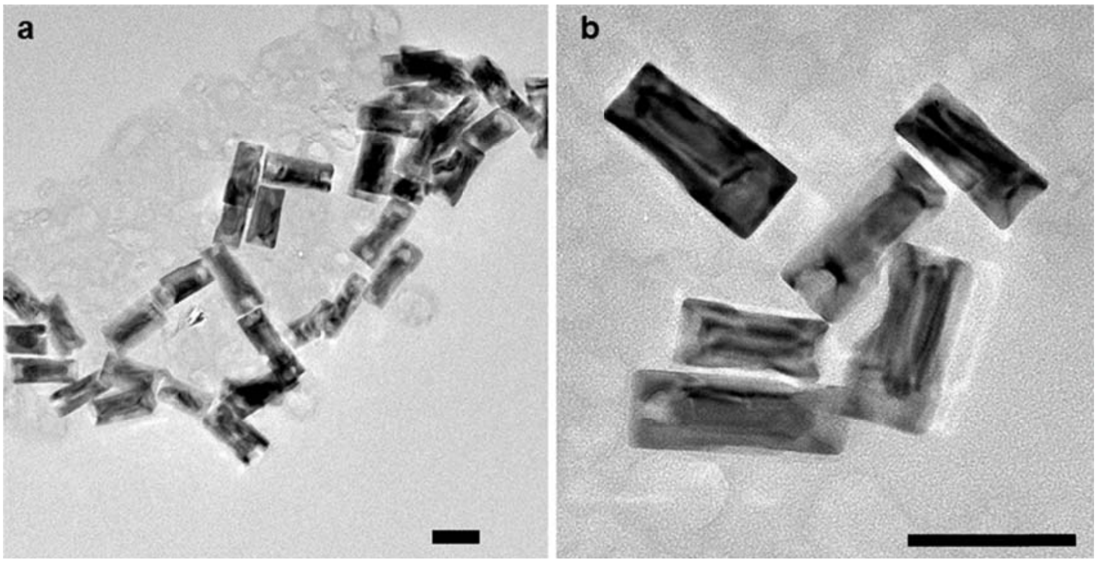

Supplementary Figure 3 | TEM images of isolated coaxial-like Au-Pd MCNs after the dissociation of the MCN chains in DMF. Both the coaxial structure and the morphology of the shell were well preserved after solvent exchange. Scale bars are 50 nm.

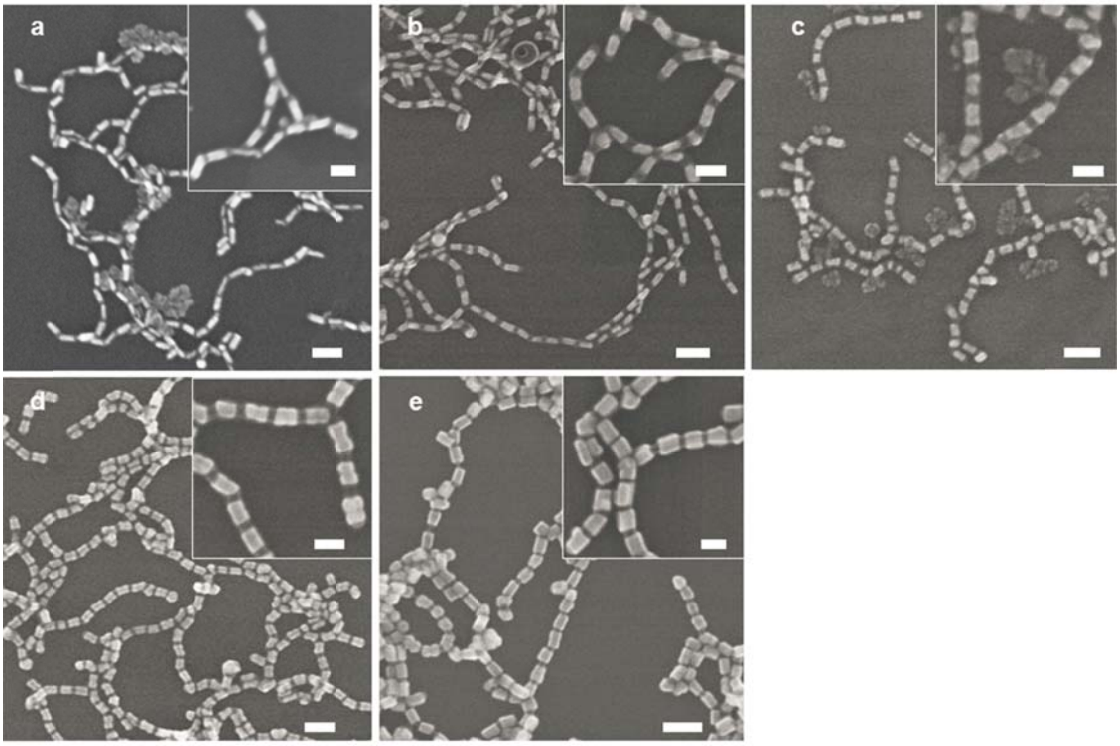

Supplementary Figure 4 | SEM images of chains of coaxial-like Au-Pd MCNs with different Pd shell thicknesses. The MCNs were synthesized by using (**a**) 0.1 mL, (**b**) 0.2 mL, (**c**) 0.4 mL, (**d**) 0.8 mL and (**e**) 1.6 mL of 10 mM H_2PdCl_4 aqueous solution as precursors in the process of Pd shell growth. Detailed synthesis information was given in Supplementary Methods. Insets are corresponding HRSEM images. Scale bars are 100nm in **a-e** and 50 nm in insets of **a-e**.

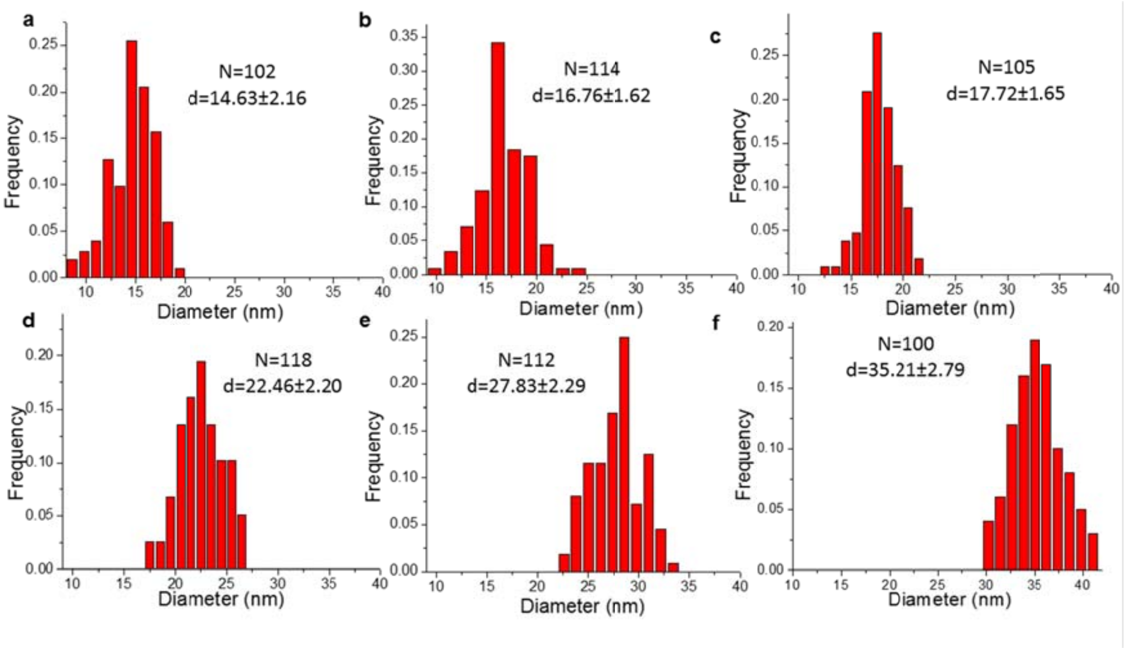

Supplementary Figure 5 | Histograms of diameter distribution of coaxial-like Au-Pd MCNs with different shell thicknesses. (**a**) Pristine Au nanorods, (**b-f**) Corresponding diameter distribution of coaxial-like Au-Pd MCNs shown in Supplementary Fig. 4a-e, respectively. Mean diameter (*d*, nm) and number of MCNs counted (*N*) for each case were given as insets.

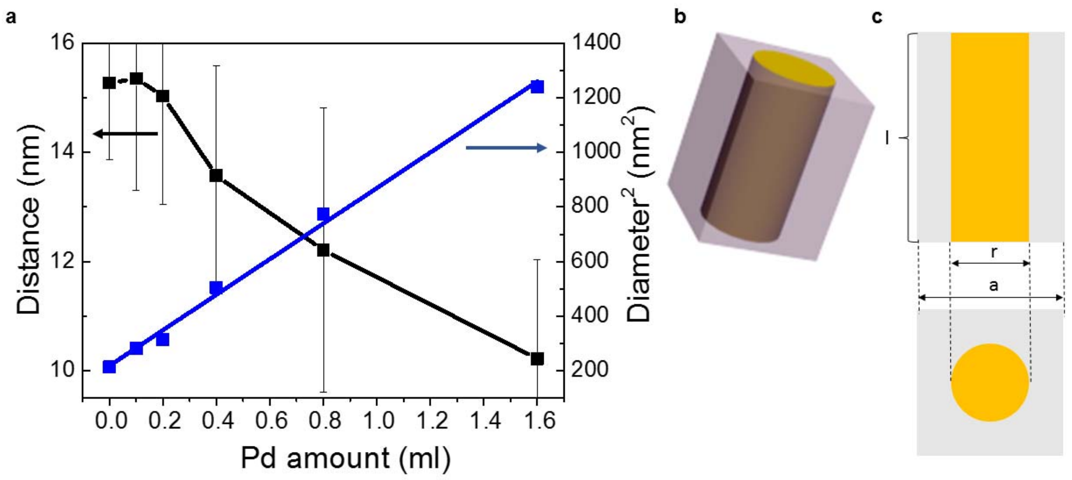

Supplementary Figure 6 | Plot of end-to-end distance and square of diameter of chains of coaxial-like MCNs as a function of Pd precursor amount and simplified coaxial-like MCN model for estimation. (**a**) Plot of end-to-end distance and square of diameter of chains of coaxial-like MCNs as a function of Pd precursor amount. (**b**) Simplified structural model of coaxial-like MCN and (**c**) corresponding side view and top view. Detailed estimation of the scaling principle of end-to-end distance and diameter with increasing Pd precursor amount was given in Supplementary Methods.

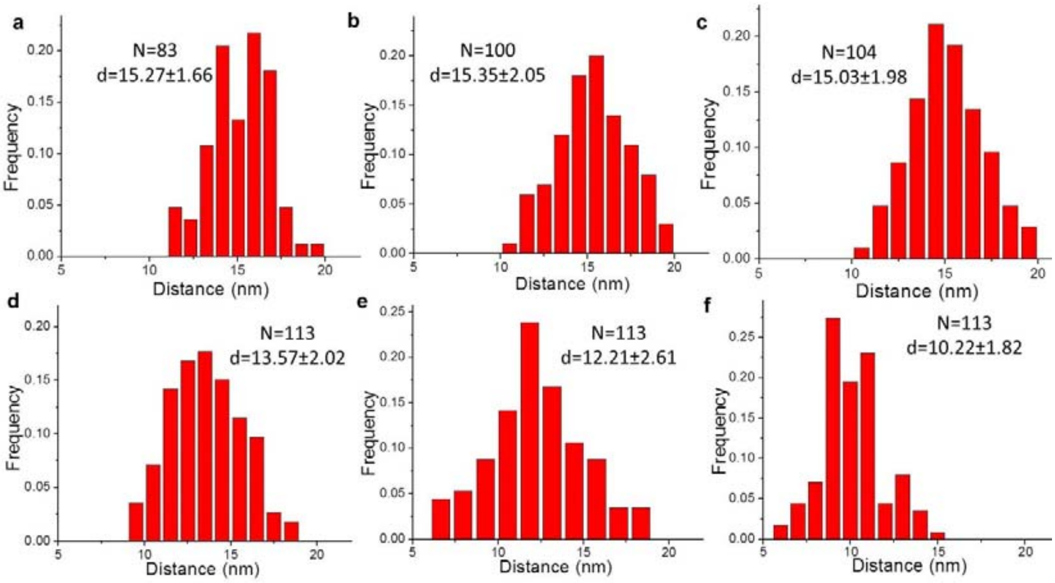

Supplementary Figure 7 | Histograms of end-to-end distance distribution of coaxial-like Au-Pd MCNs with different shell thicknesses. (**a**) Pristine Au nanorods, (**b-f**) Corresponding end-to-end distance distribution of coaxial-like Au-Pd MCNs shown in Supplementary Fig. 4a-e respectively. Mean distance (*d*, nm) and number of MCNs counted (*N*) for each case were given as insets.

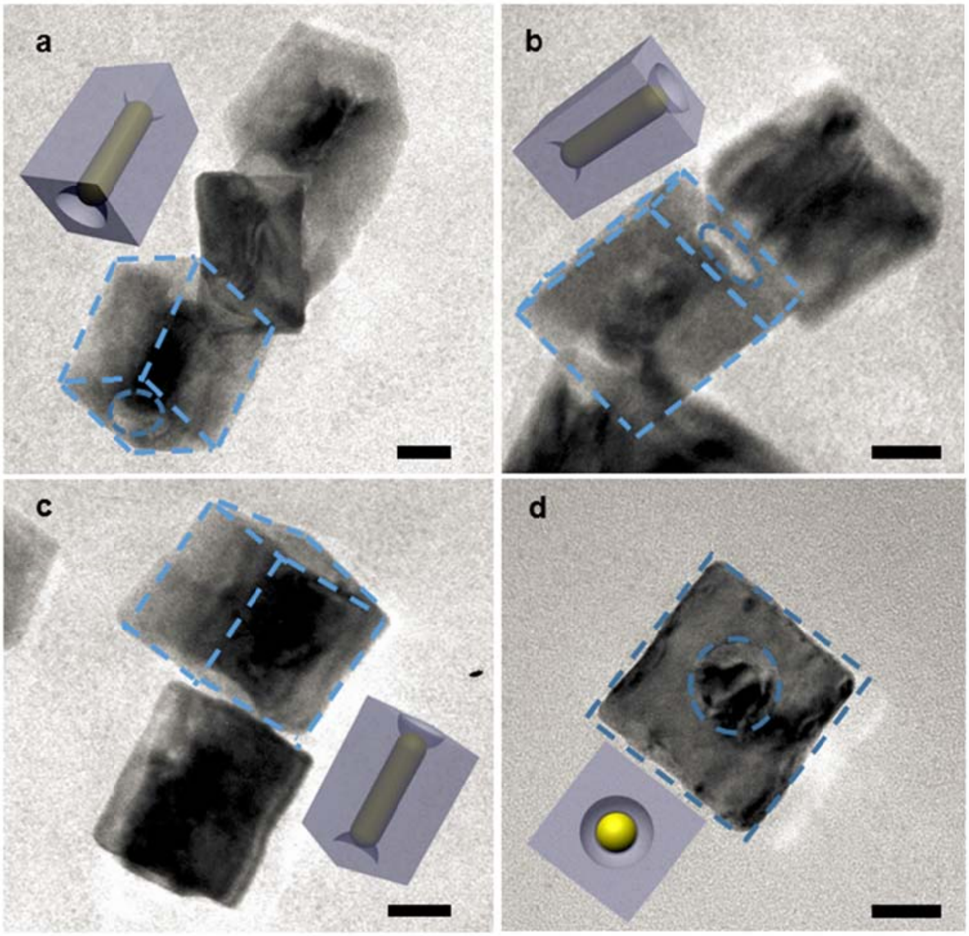

Supplementary Figure 8 | HRTEM images of tilted coaxial-like Au-Pd MCNs showing three dimensional view of the cavities at the Au narorod tips. (**a**-**c**) Coaxial-like Au-Pd MCNs with different orientations and (**d**) coaxial-like Au-Pd MCN from top-down view. Dashed Blue curves outline the cuboid morphology and cavities at the tip. Insets are corresponding structural models. All scale bars are 20 nm.

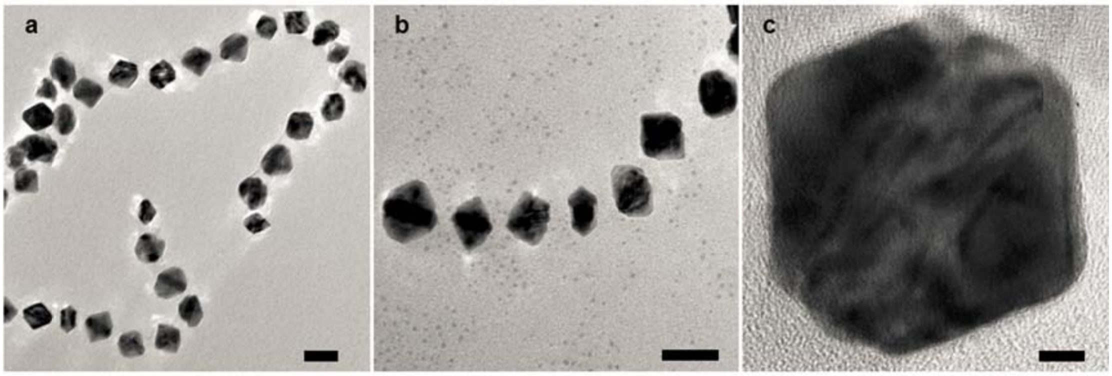

Supplementary Figure 9 | TEM images of coaxial-like Au-Pd MCNs with truncated octahedron shell. The octahedron shell was truncated at two opposite tips to expose the tips of Au nanorods (as highlighted in **c**).The formation of octahedron shell was achieved by introducing extra HCl in the solution. As discussed in previous literature, HCl together with dissolved oxygen facilitated the oxidative etching of Pd, while oxidized Pd^{2+} would be reduced and deposited back to seeds by AA in the growth solution. The octahedron shaped shell was thus formed as a consequence of synergetic effect of etching and regrowth of Pd. Detailed synthesis information was given in Supplementary Methods. Scale bars are 100 nm in **a**, **b**, and 5 nm in **c**.

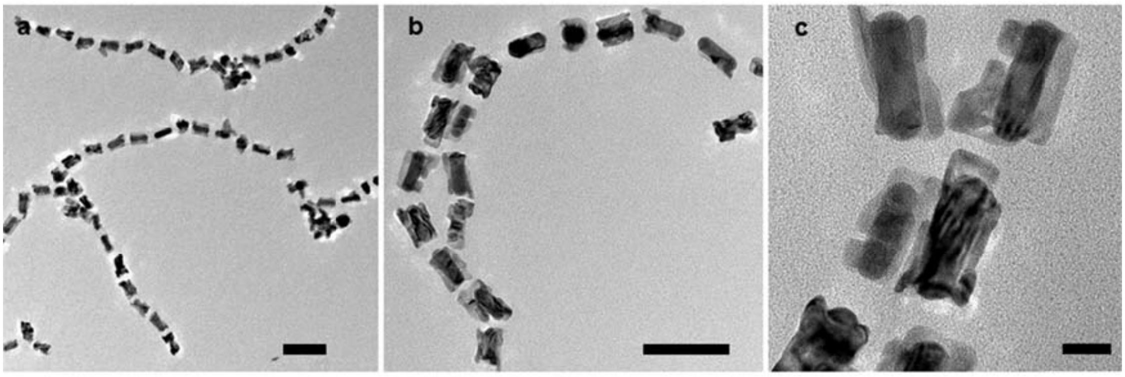

Supplementary Figure 10 | TEM images of coaxial-like Au-Pd MCNs with jagged shell.

Na_2PdCl_4 was used as Pd precursor instead of H_2PdCl_4 for the preparation of coaxial-like Au-Pd MCNs with jagged shell. Detailed synthesis information was given in Supplementary Methods. Scale bars are 100 nm in **a**, **b**, and 20 nm in **c**.

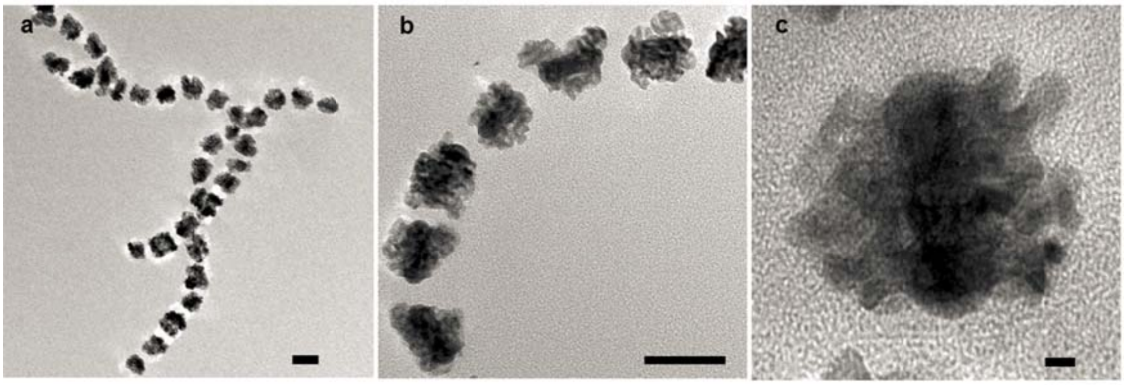

Supplementary Figure 11｜ TEM images of coaxial-like Au-Pd MCNs with porous Pd shell.

CTAC instead of CTAB was used as surfactant for the preparation of coaxial-like Au-Pd MCNs with porous shell. Detailed synthesis information was given in Supplementary Methods. Scale bars are 50 nm in **a**, **b**, and 5 nm in **c**.

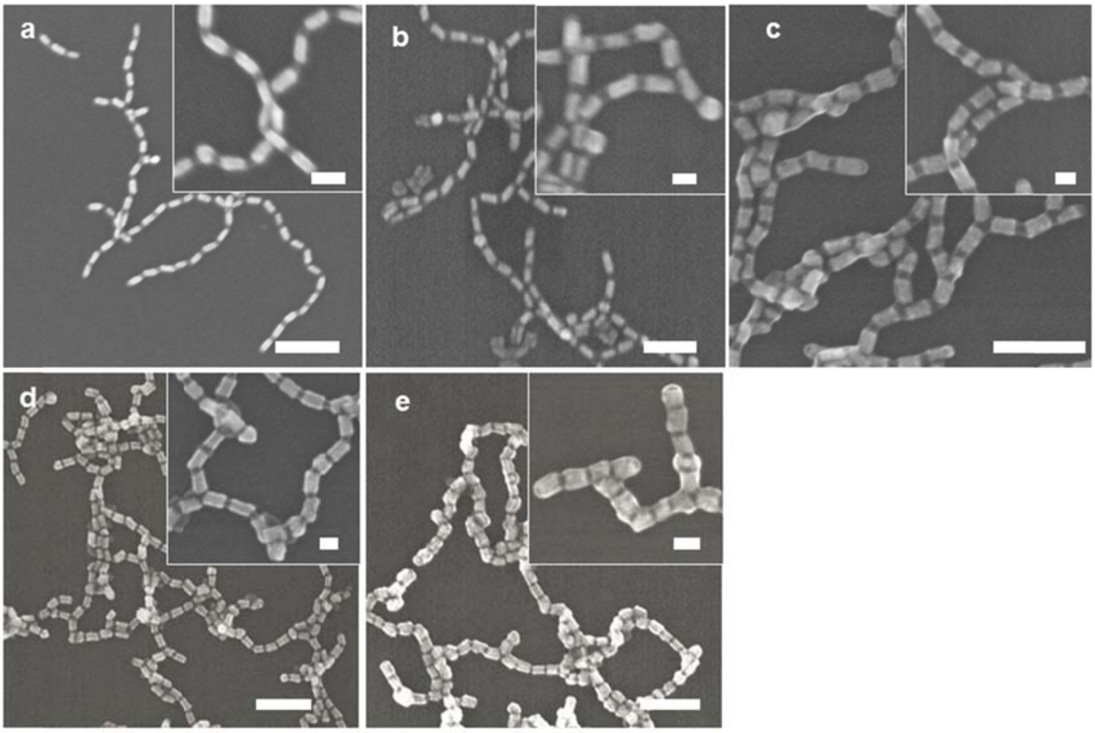

Supplementary Figure 12 | SEM images of coaxial-like Au-Pd MCNs with porous shell with different shell thicknesses. The MCNs were synthesized by using (**a**) 0.1 mL, (**b**) 0.2 mL, (**c**) 0.4 mL, (**d**) 0.8 mL and (**e**) 1.6 mL of 10 mM H_2PdCl_4 aqueous solution as precursors in the process of porous Pd shell growth. Detailed synthesis information was given in Supplementary Methods. Insets are corresponding HRSEM images. Scale bars are 200 nm in **a**-**e** and 50 nm in insets of **a**-**e**.

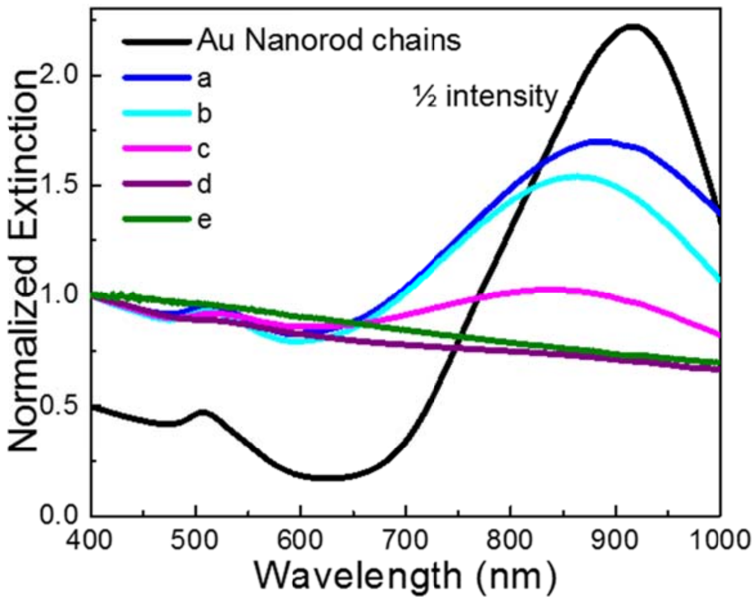

Supplementary Figure 13 | Normalized extinction spectrum of chains of coaxial-like Au-Pd MCNs with porous shell with different shell thicknesses. Black curve shows the extinction spectrum of Au nanorod chains. Curve **a-e** are corresponding extinction spectrum of chains of coaxial-like Au-Pd MCNs shown in Supplementary Fig. 12a-e respectively. Significant LSPR peak damping upon Porous Pd coating were observed similar to that of coaxial-like Au-Pd MCNs with dense cuboid shell (Fig. 2i).

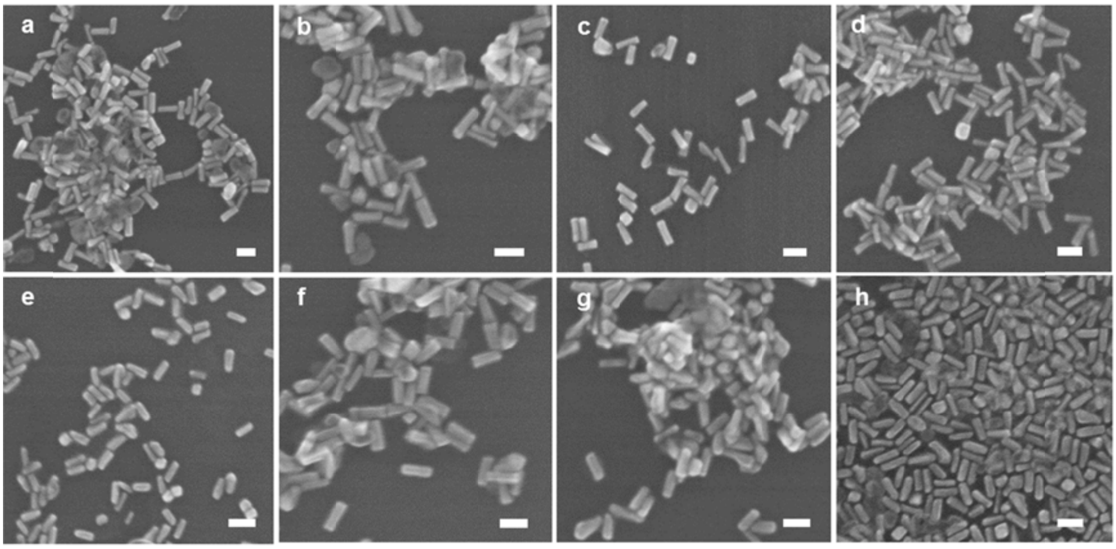

Supplementary Figure 14 | SEM images showing the formation of core-shell type Au@Pd MCNs upon the Pd deposition on PEO-tethered Au nanorods. The MCNs were synthesized by using (**a**, **e**) 0.5, (**b**, **f**) 1, (**c**, **g**) 2 and (**d**, **g**) 5 times amount PEO (of that used in PS modification) modified Au nanorods as seeds, and by using (**a**-**d**) 0.3 ml and (**e**-**h**) 0.8 ml of H_2PdCl_4 aqueous solution as precursors in the process of Pd shell growth. All MCNs exhibit cuboid morphology, which was similar to the structure derived from Pd growth on pure CTAB-capped Au nanorods[1]. Detailed synthesis information was given in Supplementary Methods. All scale bars are 50 nm.

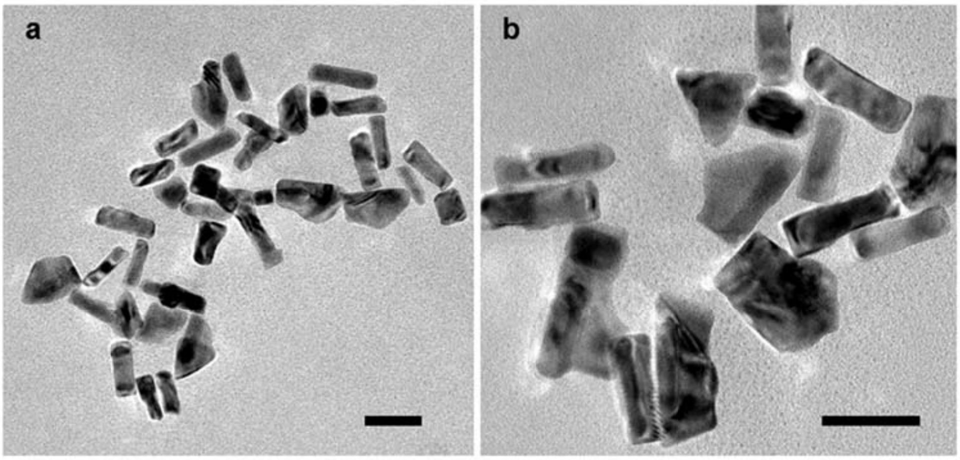

Supplementary Figure 15 | TEM images of core-shell type Au@Pd MCNs synthesized using Au nanorods with highest PEO graft density as seeds. (**a**) TEM and (**b**) HRTEM images of the core-shell type Au@Pd MCNs shown in Supplementary Fig. 14h. A typical core-shell structure with Au nanorod embedding in continuous Pd shell was observed, as hydrophilic PEO was incapable of blocking the Pd deposition on the surface of Au nanorod seeds. The anisotropic Pd growth might arise from uniform PEO coverage on Au nanorod surfaces[2]. Scale bars are 50 nm.

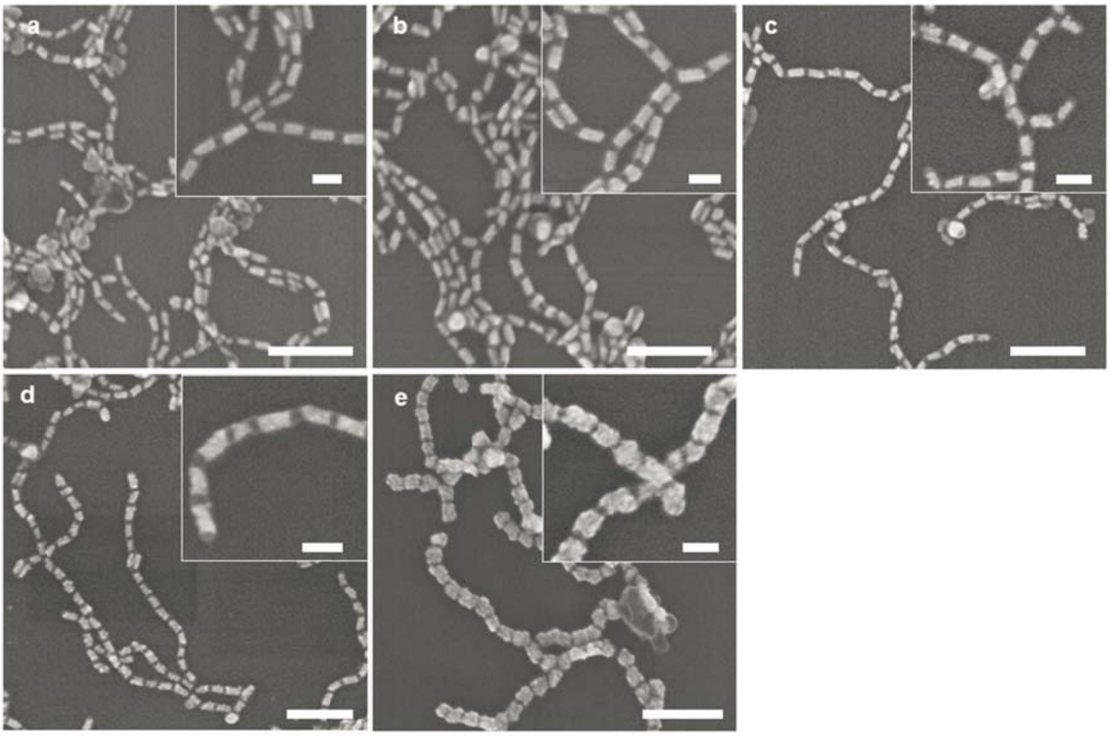

Supplementary Figure 16 | SEM images of coaxial-like Au-Pt MCNs with different shell thicknesses. The MCNs were synthesized by using (**a**) 0.1 mL, (**b**) 0.2 mL, (**c**) 0.4 mL, (**d**) 0.8 mL and (**e**) 1.6 mL of 10 mM K_2PtCl_4 aqueous solution as precursors in the process of Pt shell growth. Detailed synthesis information was given in Supplementary Methods. Scale bars are 200 nm in **a**-**e** and 50 nm in insets of **a**-**e**.

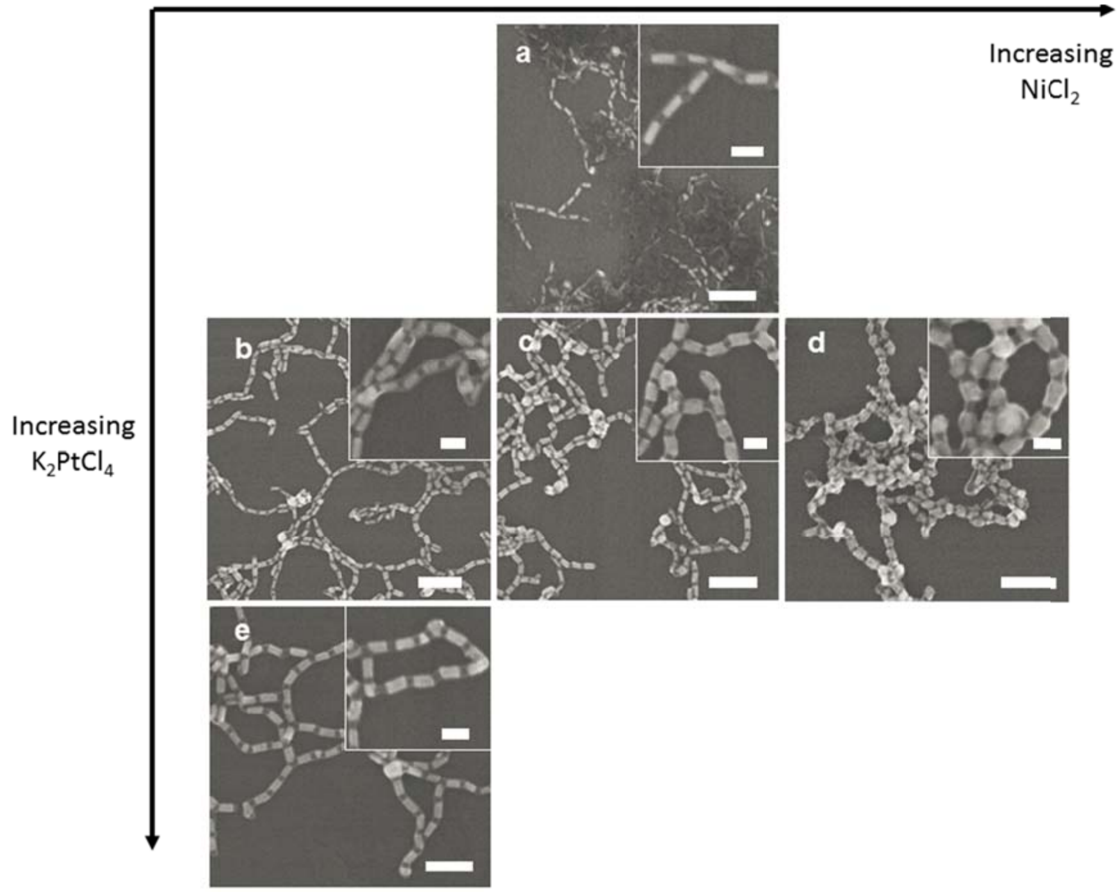

Supplementary Figure 17 | SEM images of coaxial-like Au-Pt/Ni MCNs with various shell compositions. The MCNs were synthesized by using (**a**) 0.3 ml $NiCl_2$, (**b**) 0.1 ml K_2PtCl_4 and 0.1 ml $NiCl_2$, (**c**) 0.1 K_2PtCl_4 ml and 0.3 ml $NiCl_2$, (**d**) 0.1 ml K_2PtCl_4 and 0.6 ml $NiCl_2$, (**e**) 0.3 ml K_2PtCl_4 and 0.1 ml $NiCl_2$ aqueous solution as precursors in the process of Pt/Ni shell growth. Detailed synthesis information was given in Supplementary Methods. Scale bars are 200 nm in **a**-**e** and 50 nm in insets of **a**-**e**.

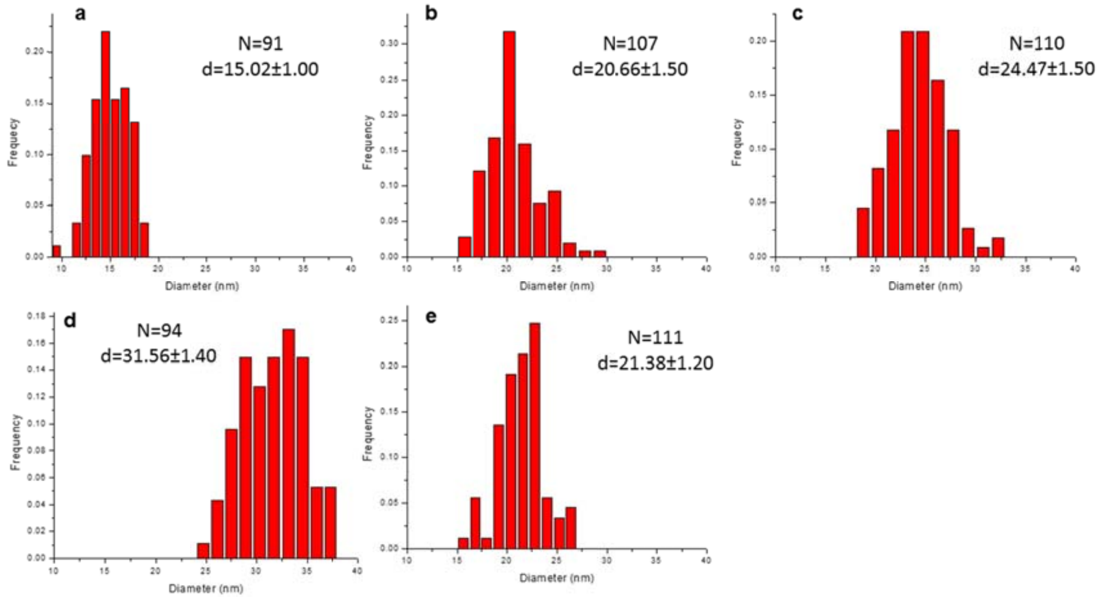

Supplementary Figure 18 | Histograms of diameter distribution of coaxial-like Au-Pt/Ni MCNs with various shell compositions. (**a**-**e**) Diameter distribution of coaxial-like Au-Pt/Ni MCNs shown in Supplementary Fig. 20a-e respectively. Mean diameter (*d*, nm) and number of MCNs counted (*N*) for each case were given as insets.

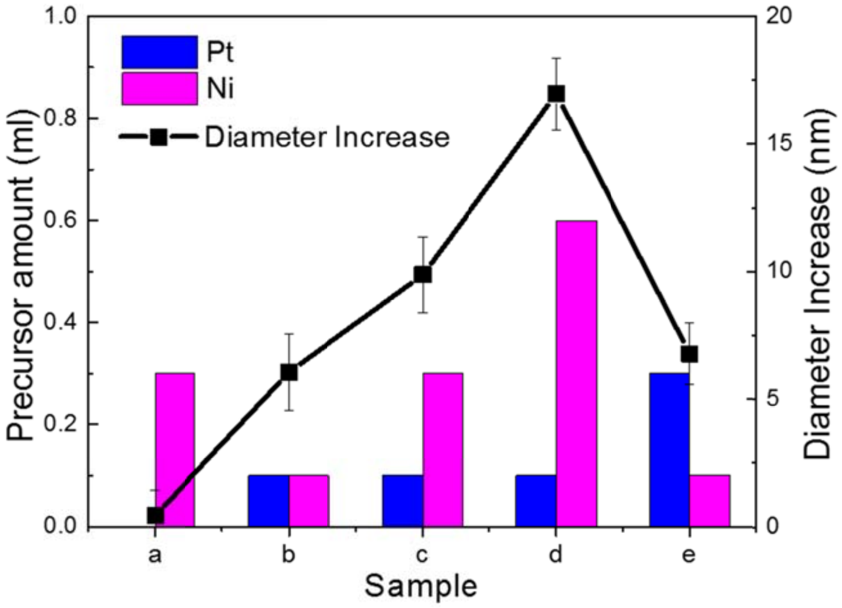

Supplementary Figure 19 | **Plot of Pt/Ni precursor amount and diameter increase of coaxial-like Au-Pt/Ni MCNs with various shell compositions.** A comparison between sample **a** and **c** shows that the diameter of MCNs did not change when Ni precursor was used in the absence of Pt precursor, indicating that Ni would not be reduced without Pt. This is in accordance with previous finding that Pt is pivotal in catalytic reduction of Ni[3]. The diameter of coaxial-like Au-Pt/Ni MCNs increased linearly with increasing the amount of Ni precursor in the presence of Pt precursor (**b**-**d**), while it did not change significantly with increasing the amount of Pt precursor (**b**, **e**).

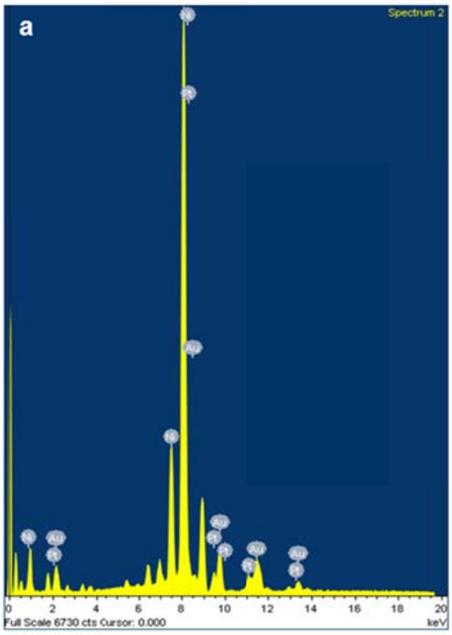

b

Element	Weight%	Atomic%
Ni	27.87	56.37
Pt	21.60	13.15
Au	50.53	30.47
Total	100.00	

Supplementary Figure 20 | EDS elemental analysis and corresponding elemental composition of coaxial-like Au-Pt/Ni MCNs. (**a**) Single point EDS elemental analysis of the sample shown in Supplementary Fig. 20c. (**b**) Corresponding elemental composition of coaxial-like Au-Pt/Ni MCNs. Elemental analysis further confirmed the successful reduction of Pt and Ni. The Ni: Pt ratio is around 4.3:1, which is slightly larger than the feeding ratio of Ni: Pt precursors (3:1).

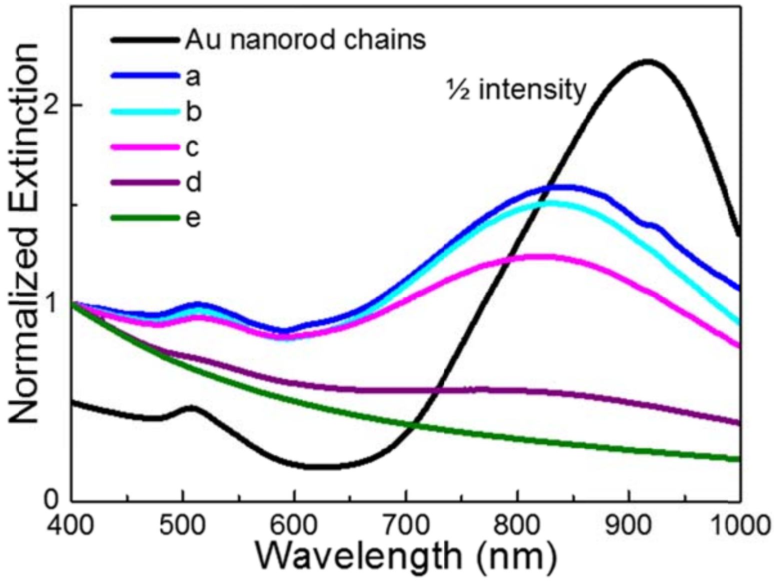

Supplementary Figure 21 | Normalized extinction spectrum of coaxial-like Au-Pt MCNs with different shell thicknesses. Black curve shows the extinction spectrum of Au nanorod chains. Curve **a-e** are corresponding extinction spectrum of chains of coaxial-like Au-Pt MCNs shown in Supplementary Fig. 16a-e, respectively. Significant LSPR peak damping upon Pt shell growth were observed similar to that of coaxial-like Au-Pd MCNs.

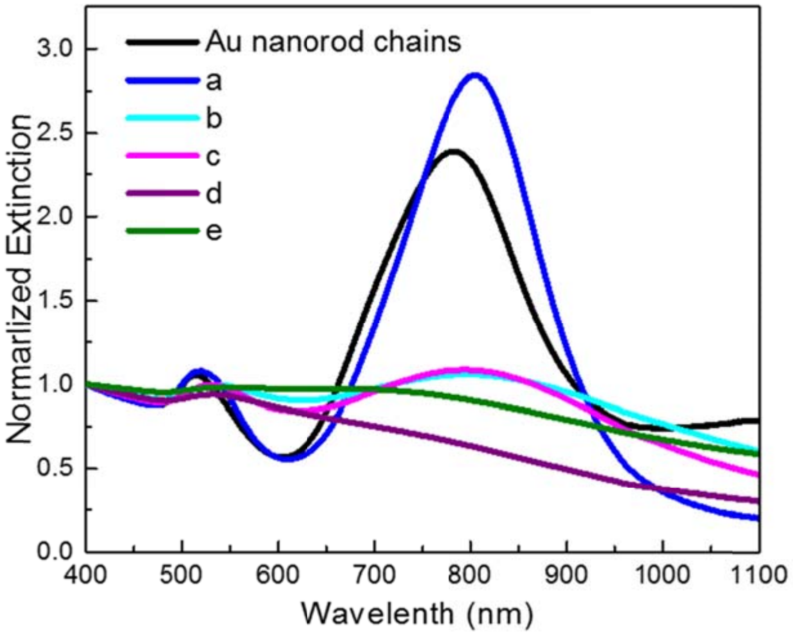

Supplementary Figure 22 | Normalized extinction spectrum of coaxial-like Au-Pt/Ni MCNs with various shell compositions. Black curve shows the extinction spectrum of Au nanorod chains. Curve **a-e** are corresponding extinction spectrum of chains of coaxial-like Au-Pt/Ni MCNs shown in Supplementary Fig. 18a-e, respectively.

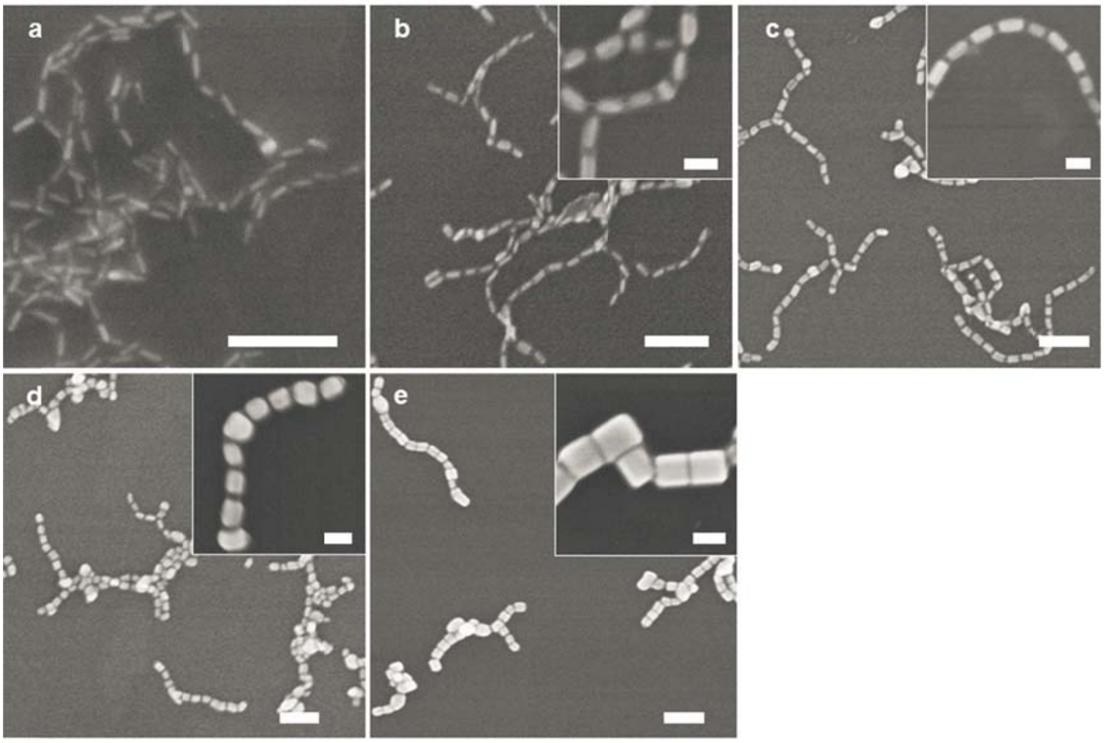

Supplementary Figure 23 | SEM images of coaxial-like Au-Ag MCNs with different shell thicknesses. The MCNs were synthesized by using (**a**) 0.1 mL, (**b**) 0.2 mL, (**c**) 0.4 mL, (**d**) 0.8 mL and (**e**) 1.6 mL of 10 mM $AgNO_3$ aqueous solution as precursors in the process of Ag shell growth. Detailed synthesis information was given in Supplementary Methods. Scale bars are 200 nm in **a**-**e** and 50 nm in insets of **a**-**e**.

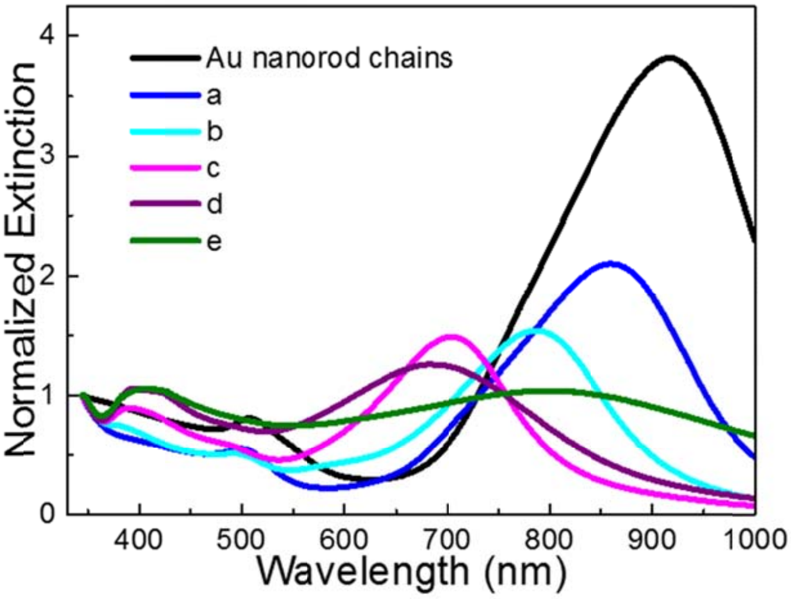

Supplementary Figure 24 | Normalized extinction spectrum of chains of coaxial-like Au-Ag MCNs with different shell thicknesses. Black curve shows the extinction spectrum of Au nanorod chains. Curve **a-e** are corresponding extinction spectrum of chains of coaxial-like Au-Ag MCNs shown in Supplementary Fig. 23a-e respectively. Ag growth initially led to gradual blue shift of the longitudinal and transverse LSPR peak from 920 nm and 510 nm to 690 nm and 400 nm respectively, as seen from curve **a-d**. This can be explained by changes of effective dielectric functions and aspect ratio of the overall MCNs with increasing Ag shell thickness[2, 4]. However, the further growth of Ag resulted in significant red shift of longitudinal LSPR back to around 800 nm, due to the arising of strong longitudinal LSPR coupling with dramatically decreased end-to-end distance (curve **e**).

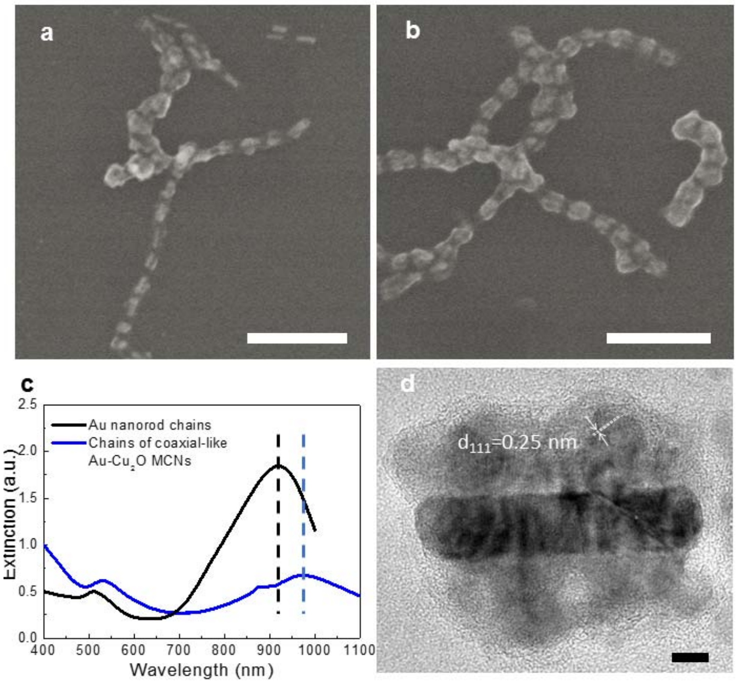

Supplementary Figure 25 | Electron microscope images and UV-Vis extinction spectrum of coaxial-like Au-Cu_2O MCNs. (**a**, **b**) SEM images of chains of coaxial-like Au-Cu_2O MCNs with thin (**a**) and thick (**b**) shell. (**c**) Extinction spectrum of Au nanorod chains and chains of coaxial-like Au-Cu_2O MCNs. The coating of Au nanorod with Cu_2O led a dramatic red shift of LSPR, as a result of the synergetic optical response of Au and Cu_2O. (**d**) HRTEM images of an individual coaxial-like Au-Cu_2O MCN showing the typical lattice plane of cubic Cu_2O (111) facet. Detailed synthesis information was given in Supplementary Methods. Scale bars are 200 nm in **a**, **b**, and 5 nm in **d**.

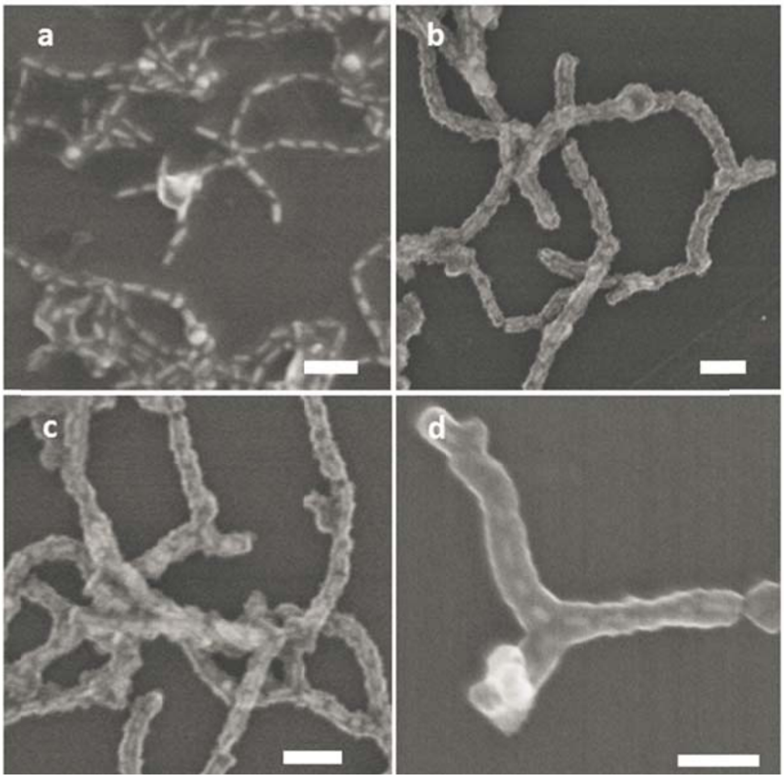

Supplementary Figure 26 | SEM images of coaxial-like Au-CeO_2 MCNs with different thicknesses. The MCNs were synthesized by using (**a**) 0.2 ml, (**b**) 0.175 ml, (**c**) 0.15 ml and (**e**) 0.1 ml of the Au nanorod chain aqueous solution as seeds in the process of CeO_2 shell growth. Detailed synthesis information was given in Supplementary Methods. CeO_2 tend to grow into continuously lays and cover the whole chain, as seen in **b-e**. All scale bars are 100 nm.

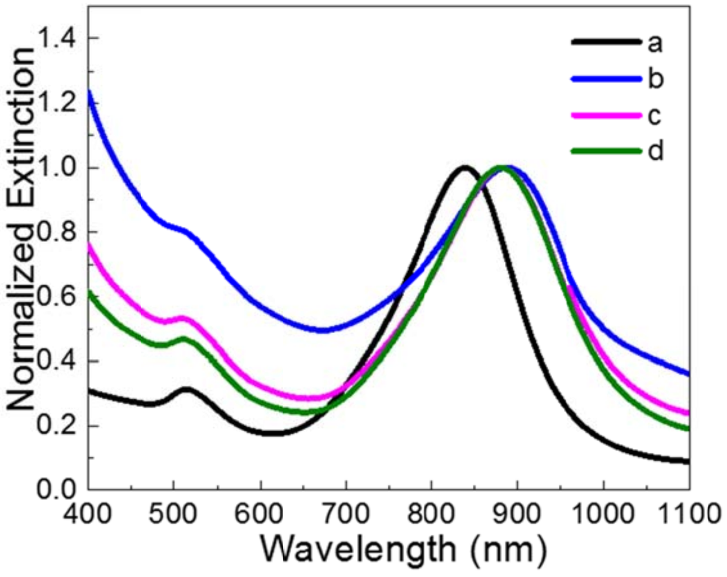

Supplementary Figure 27 | Normalized extinction spectrum of coaxial-like Au-CeO_2 MCNs with different thickness. Curve **a-e** are corresponding extinction spectrum of chains of coaxial-like Au-CeO_2 MCNs shown in Fig. 26a-d respectively. Coating of CeO_2 also lead to a slight red shift of LSPR, due to the large refractive index of CeO_2 compared with water.

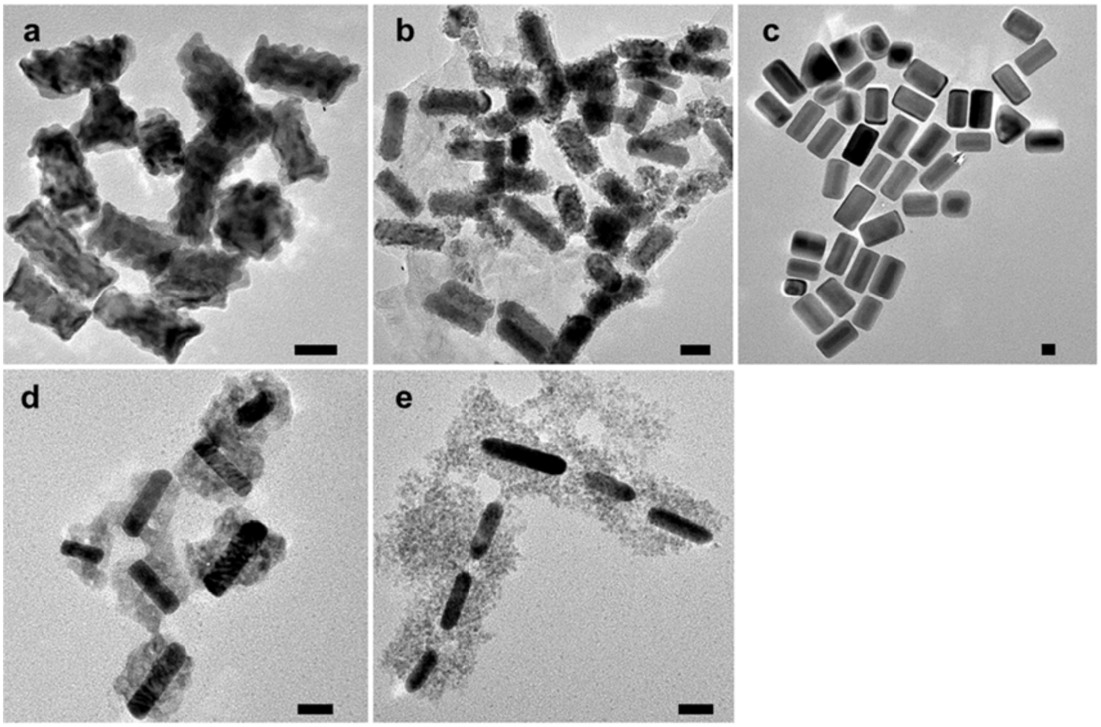

Supplementary Figure 28 | TEM images of dissociated coaxial-like MCNs with different shell materials: (**a**) Pt, (**b**) Pt-Ni, (**c**) Ag, (**d**) Cu_2O and (**e**) CeO_2. The random orientation of those MCNs indicates that they are dissociated into individual MCNs. Polymers are solubilized to stabilize the MCNs in organic solvents. Some MCNs still maintained chain-like structure in the case of CeO_2 coating, due to the formation of continuous CeO_2 shell. All scale bars are 20 nm.

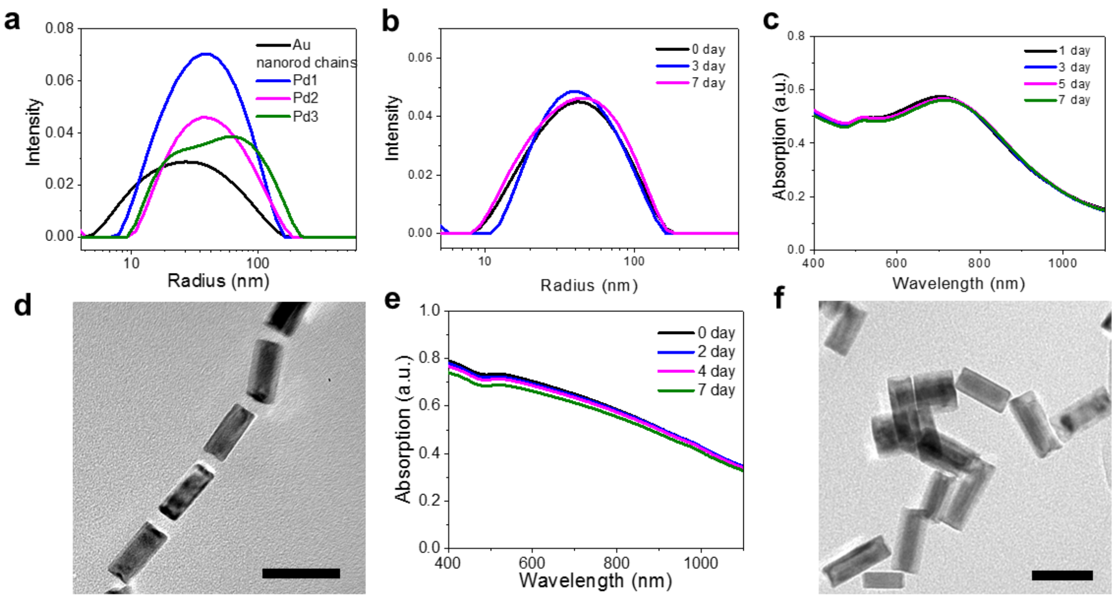

Supplementary Figure 29 | **Stability test of coaxial-like Au-Pd MCNs in solution.** (**a**) DLS spectrum of Au nanorod chains and Au-Pd coaxial-like MCN chains with different shell thicknesses. The hydrodynamic radius gradually increases from ~37 nm of Au nanorod chains to 47nm, 51 nm and 62 nm with increasing Pd shell thickness. (**b**) Time-dependent DLS spectrum of Au-Pd coaxial-like MCN chains, with an average hydrodynamic radius of ~51 nm. (**c**)Time-dependent UV-Vis spectrum of Au-Pd coaxial-like MCN chains. (**d**) Representative TEM image of a single Au-Pd coaxial-like MCN chain 7 days after preparation. (**e**) Time-dependent UV-Vis spectrum of isolated individual Au-Pd coaxial-like MCNs and (**f**) Representative TEM image of isolated individual Au-Pd coaxial-like MCNs 7 days after preparation. All scale bars are 50 nm.

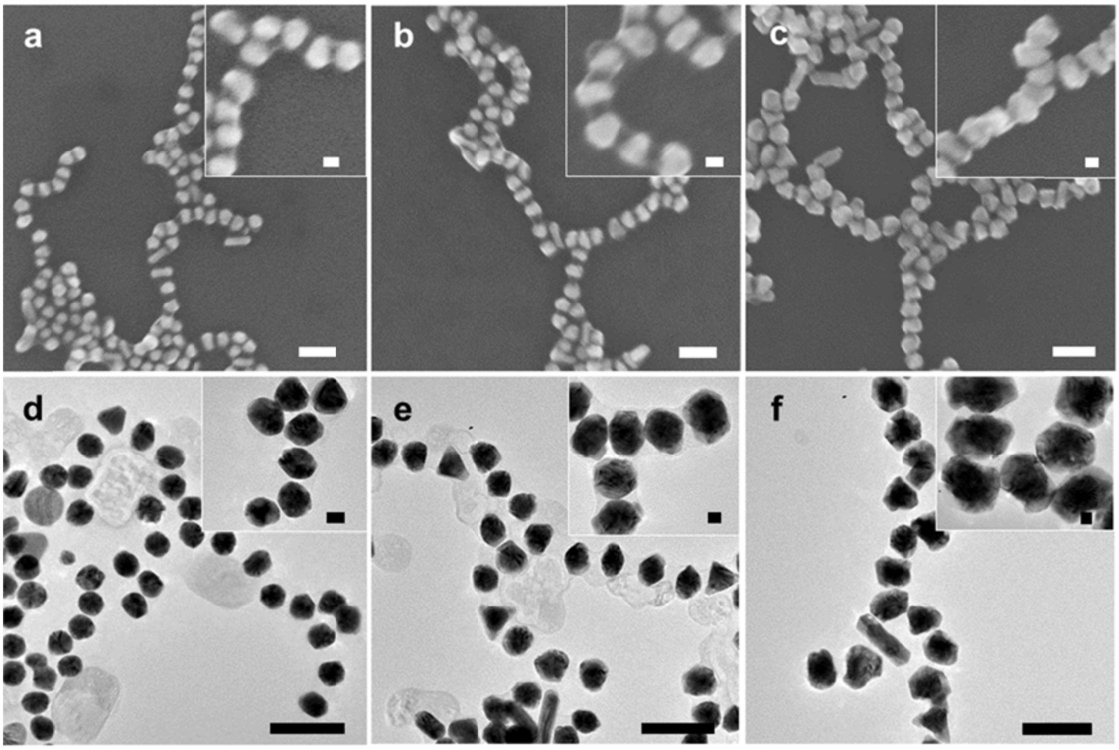

Supplementary Figure 30 | SEM and TEM images of chains of Saturn-like Au-Pd MCNs with different shell thicknesses. The MCNs were synthesized by using (**a**, **d**) 0.1 mL, (**b**, **e**) 0.2 mL and (**c**, **f**) 0.4 mL of 10 mM H_2PdCl_4 aqueous solution as precursors in the process of Pd shell growth. Detailed synthesis information was given in Supplementary Methods. Scale bars are 100 nm in **a**-**f** and 10 nm in insets of **a**-**f**.

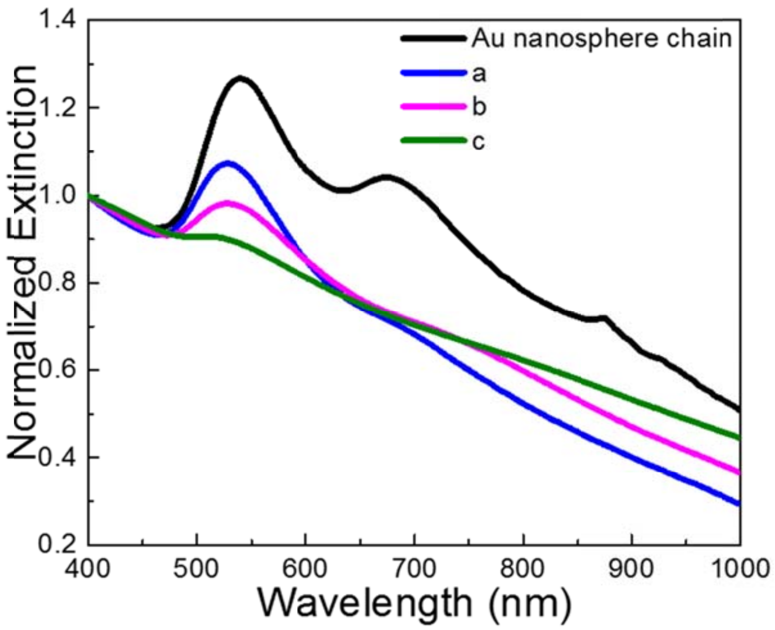

Supplementary Figure 31 | Normalized extinction spectrum of chains of Saturn-like Au-Pd MCNs with different shell thicknesses. Black curve shows the extinction spectrum of Au nanosphere chains. Curve **a-c** are corresponding extinction spectrum of chains of Saturn-like Au-Pd MCNs shown in Supplementary Fig. 29a-c respectively. Au nanosphere chains exhibit two characteristic plasmonic peaks at 540 nm and 670 nm, which could be attributed to the original LSPR of Au nanospheres and longitudinal surface plasmon coupling of the one dimensional structure respectively. Significant LSPR peak damping upon Pd coating was observed similar to that of coaxial-like Au-Pd MCNs. Moreover, the peak at 670 nm totally disappeared, indicating that the coupling was significantly weakened as a result of the LSPR damping.

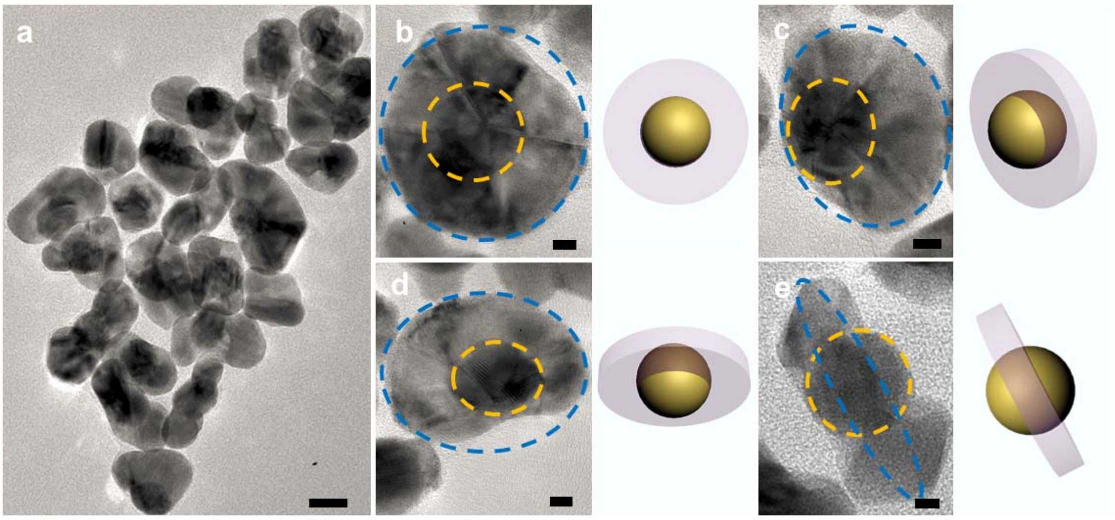

Supplementary Figure 32 | TEM images of dissociated Saturn-like Au-Pd MCNs of different orientations. (**a**) TEM image of dissociated Saturn-like Au-Pd MCNs showing a large area. (**b-e**) HRTEM images of dissociated individual Au-Pd MCNs of different orientations with gold circles highlighting the Au core and blue circles outlining the shell. Corresponding structural models were shown aside. Scale bars are 20 nm in **a**, and 5 nm in **b-e**.

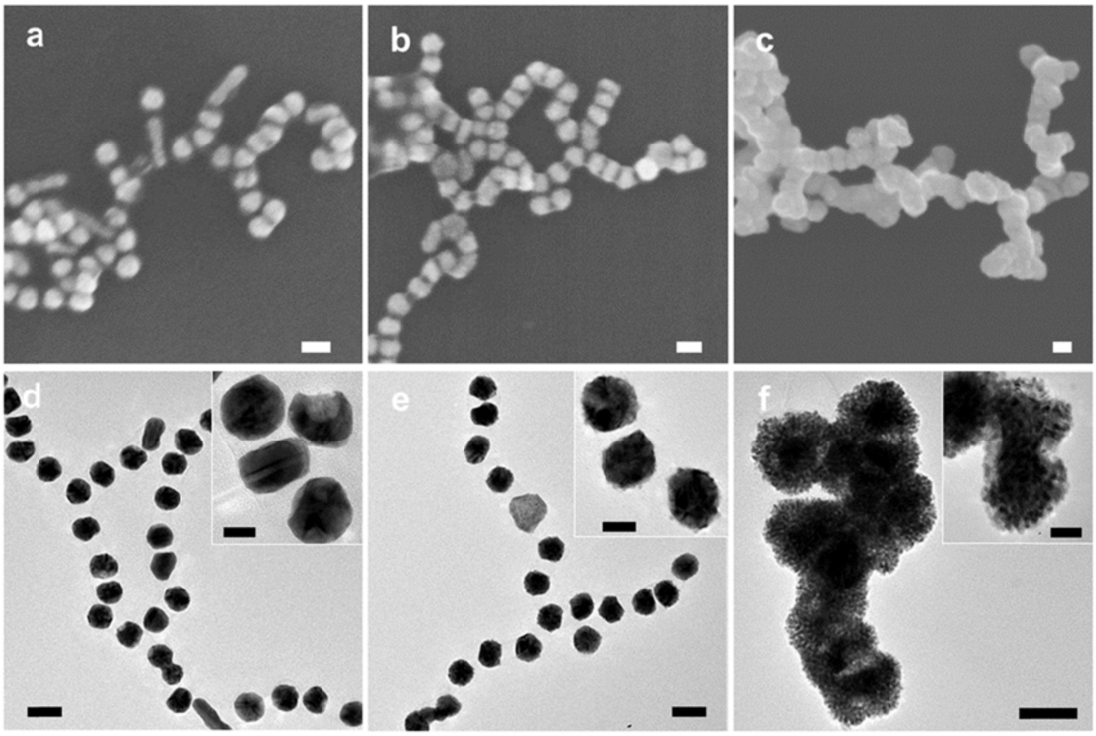

Supplementary Figure 33 | SEM and TEM images of chains of Saturn-like Au-Pt MCNs with different shell thicknesses. The MCNs were synthesized by using (**a**, **d**) 0.1 mL, (**b**, **e**) 0.2 mL and (**c**, **f**) 0.4 mL of 10 mM K_2PtCl_4 aqueous solution as precursors in the process of Pt shell growth. Detailed synthesis information was given in Supplementary Methods. Scale bars are 50 nm in **a**-**f** and 10 nm in insets of **d**-**f**.

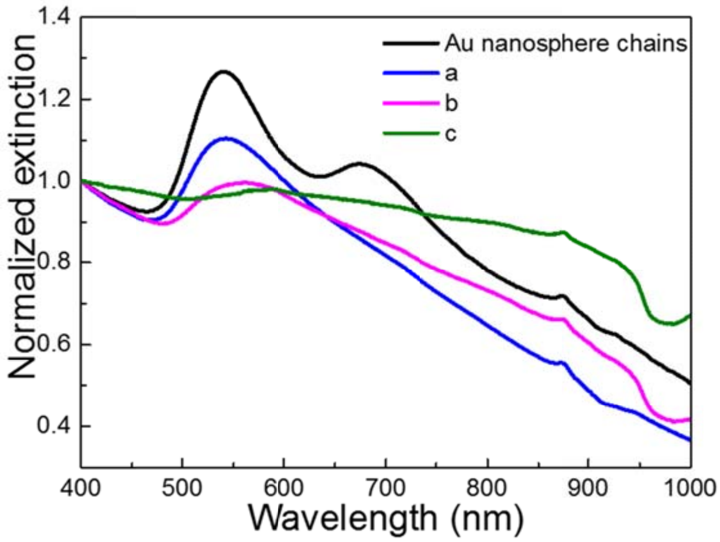

Supplementary Figure 34 | Normalized extinction spectrum of chains of Saturn-like Au-Pt MCNs with different shell thickness. Black curve shows the extinction spectrum of Au nanosphere chains. Curve **a-c** are corresponding extinction spectrum of chains of Saturn-like Au-Pt MCNs shown in Supplementary Fig. 32a-c respectively. Similar damping of the two surface plasmonic peaks of was observed as in the case of Saturn-like Au-Pd MCN chains.

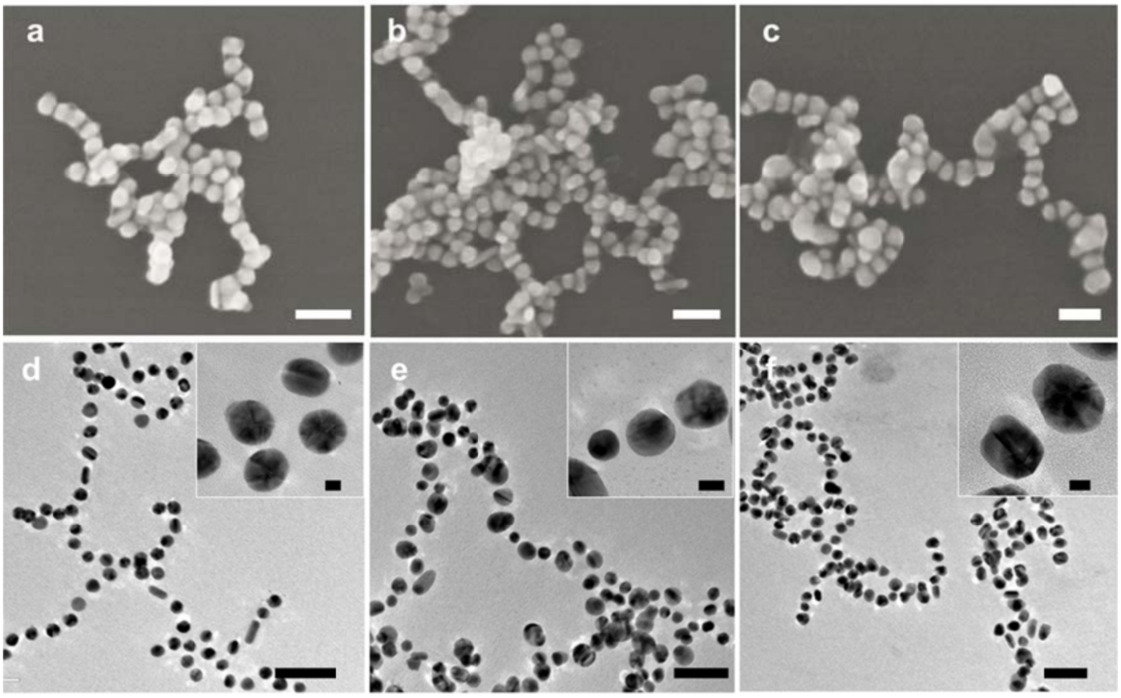

Supplementary Figure 35 | SEM and TEM images of chains of Saturn-like Au-Ag MCNs with different shell thicknesses. The MCNs were synthesized by using (**a**, **d**) 0.1 mL, (**b**, **e**) 0.2 mL and (**c**, **f**) 0.4 mL of 10 mM $AgNO_3$ aqueous solution as precursors in the process of Ag shell growth. Detailed synthesis information was given in Supplementary Methods. Scale bars are 100 nm in **a**-**f** and 10 nm in insets of **d**-**f**.

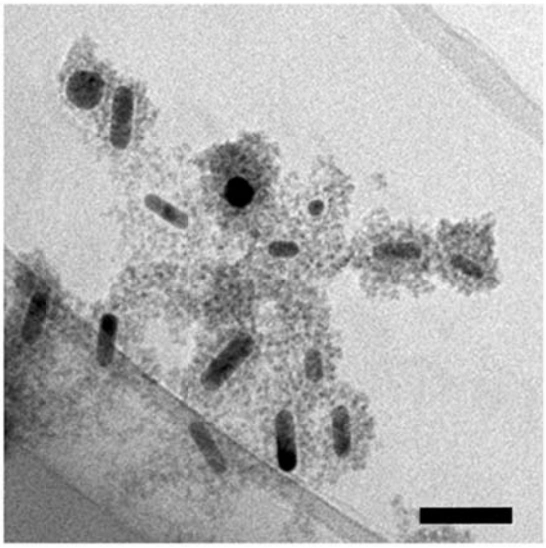

Supplementary Figure 36 | TEM image of core-shell type Au-CeO_2 MCNs.

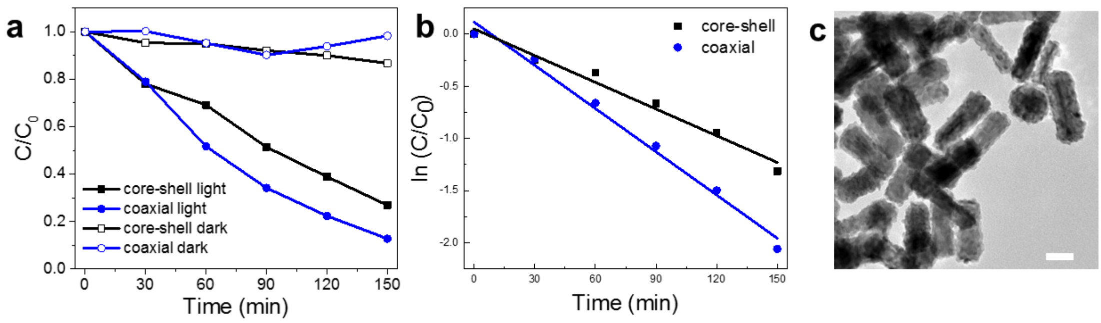

Supplementary Figure 37 | Photo degradation of MB to benzaldehyde with coaxial-like and core-shell type Au-CeO_2 MCNs. (**a**) Time-dependent profiles of photo degradation of MB with coaxial-like and core-shell type Au-Pt MCNs under visible light and in dark respectively. (**b**) Kinetic linear fitting curves for reduction of MB with coaxial and core-shell Au-Pt MCNs under light. A first-order kinetic rate constant of 0.013 min^{-1} was calculated for coaxial-like MCNs, which is 1.6 times larger than that of core-shell type MCNs (0.008 min^{-1}). (**c**) TEM image showing core-shell type Au@Pt MCNs. Scale bar, 20 nm.

Supplementary Methods

Regents

Gold(III) chloride trihydate ($HAuCl_4•3H_2O$, 99.9+%), Silver nitrate ($AgNO_3$, 99+%), Potassium tetrachloroplatinate(II) (K_2PtCl_4, 98+%), Sodium tetrachloropalladate(II) (Na_2PdCl_4, 99.9+%), Palladium(II) chloride ($PdCl_2$, 99+%), Nickel(II) chloride hexahydrate ($NiCl_2•6H_2O$, 98+%), Copper(II) nitrate trihydrate ($Cu(NO_3)_2•3H_2O$, 99%+), Cerium(III) nitrate hexahydrate ($Ce(NO_3)_3•6H_2O$, 99%+), Sodium hydroxide (NaOH, 98%+), Hydrochloric acid (HCl, 36.5%-38.0%), hydrazine (anhydrous, 98%+), Ammonium hydroxide solution (NH_4OH, 28%-30%), Ethylenediaminetetraacetic acid (EDTA, 99%+), Polyvinylpyrrolidone (PVP, M_w=29,000), Hexadecyltrimethylammonium bromide (CTAB, 99%+), Cetyltrimethylammonium chloride solution (CTAC, 25% in H_2O), Sodium borohydride ($NaBH_4$, 99.99%), L-ascorbic acid (AA, 99%+), trisodium citrate dehydrate (SC, 99.0+%), tetrahydrofuran (THF), dimethylformamide (DMF) were purchased from Sigma-Aldrich.

Undecane (99.0%+), methanol (99.5%+), methylene blue (MB, 98.5%+) and sodium bicarbonate ($NaHCO_3$, 99.5%+) were purchased from Guangfu. Toluene (99.5%+), benzyl alcohol (98.0%+) and benzaldehyde (98.5%+) were purchased from Jiangtian. Thiol-terminated polystyrene (PS-SH, M_n = 12000, M_w/M_n = 1.09) and thiol-terminated poly (ethylene oxide) (PEO-SH, M_n = 5000, M_w/M_n = 1.08) were purchased from Polymer Source Inc.

All above chemicals were used as received without further processing.

Deionized water (Millipore Milli-Q grade) with resistivity of 18.0 MΩ was used in all the experiments.

Detailed Methods

- **Synthesis CTAB capped Au nanorods**

CTAB capped Au nanorods were synthesized with a modified seed-mediated growth method[5]. A seed solution was first prepared as follows: a 2.5 ml of 0.2 M CTAB aqueous solution and a 0.125

ml of 10 mM $HAuCl_4$ aqueous solution were sequentially added into 2.375 ml of water in a 20 ml scintillation vial, followed by injection of a 0.3 ml of ice-cold 10 mM $NaBH_4$ aqueous solution in one shot under vigorous stirring. The solution immediately turned dark brown. After stirring for 2 minutes at room temperature, the solution was held in a 29 °C water bath for 2 hours before use.

The growth solution was prepared in a 250 ml conical flask. A 95 ml of 0.1 M CTAB aqueous solution, a 1 ml of 10 mM $AgNO_3$ aqueous solution, and a 5 ml of 10 mM $HAuCl_4$ aqueous solution were sequentially added to the flask, followed by injecting a 0.6 ml of 0.1 M AA aqueous solution. Upon AA injection, the flask was gently shaken till the solution turned clear. Finally, a 1.6 ml of Au seed solution was added. After gently shaking, the flask was left in a 29 °C water bath overnight.

- **Synthesis CTAB capped Au nanospheres**

CTAB capped Au nanospheres were synthesized with a modified seed-mediated growth method[6]. A seed solution was first prepared as follows: a 4.9 ml of 1mg ml^{-1} $HAuCl_4$ aqueous solution and a 3.7 ml of 1mg ml^{-1} SC aqueous solution were sequentially added into 41.4 ml of water in a 100 ml beaker under vigorous stirring. A 1.5 ml of 0.1 M ice-cold $NaBH_4$ aqueous solution was then quickly injected. The seed solution was stirred at room temperature for 3 hours.

The growth solution was prepared by sequentially adding a 7.44 ml of 15 mM $HAuCl_4$ aqueous solution and a 2.5 ml of 10 mM $AgNO_3$ aqueous solution to a 450 ml CTAB aqueous solution (containing 1.5 g CTAB) in a 1,000 ml conical flask. The flask was gently shaken after each addition. For the preparation of 20 nm Au nanospheres, a 4.6 ml of seed solution was added to the growth solution. After stirring for half an hour, the flask was left in a 29 °C water bath overnight.

- **Synthesis of coaxial-like Au-Pd MCNs with cuboid shell**

Coaxial-like Au-Pd MCNs with different shell thicknesses were synthesized as follows. In 5 different 20 ml vials, a 1 ml aqueous solution of Au nanorod chains was mixed with a 1 ml of 0.2 M CTAB aqueous solution, and diluted with 7.85 ml, 7.7 ml, 7.4 ml, 6.8 ml and 5.6 ml water,

respectively. Then 0.1ml, 0.2 ml, 0.4 ml, 0.8 ml and 1.6 ml of 10 mM H_2PdCl_4 aqueous solution were added, followed by adding 0.05 ml, 0.1 ml, 0.2 ml, 0.4 ml and 0.8 ml of 0.1 M AA aqueous solution. After gentle shaking, the vials were capped and left in a 30 °C water bath for 10 hours. The samples after growth were washed twice with water at 7000 rpm for 15 mins before characterization and further use.

- **Estimation of scaling principle of end-to-end distance and diameter of chains of Au-Pd coaxial MCNs**

The end-to-end distance of MCNs is approximately same with that of Au nanorod chains when Pd amount is small, indicating that in this case the length of Pd shell does not exceed that of Au nanorods. This is in line with our hypothesis that Pd favorably deposit on the exposed sides of Au nanorods (Fig. 1a and Fig. 2f). The distance between neighboring MCNs decreased significantly after Pd amount reached a certain threshold (Pd amount= 0.2 ml in the synthesis), where the length of Pd shell reached that of Au nanorods as a result of Pd propagation on polymer blocks. Beyond this threshold, the end-to-end distance of MCNs dramatically decrease with the gradually elongated Pd shell (Fig. 1a and Fig. 2h).

On the other hand, the dimeter of MCNs increased with increasing Pd amount. We used a simplified model to illustrate the relationship between the diameter of MCN, *R*, and the Pd precursor amount, *n*. As shown in Supplementary Fig. 7b, c, we consider the Au nanorods as ideal cylinder, and Pd forming ideal cuboid shell with length equaling to the length of Au nanorod core. The volume of Pd shell, *V*, could be given by:

$$V = l(R^2 - \pi r^2) \qquad \text{(Supplementary Equation 1)}$$

Where *l* is the length of the MCN, and *r* is diameter of Au nanorod core.

V could also be given by considering the amount of reduced Pd precursor:

$$V = \frac{nM_{Pd}}{\rho_{Pd}} \qquad \text{(Supplementary Equation 2)}$$

Where M_{Pd} is the atom weight of Pd, and ρ_{Pd} is the density of Pd shell.

Combine Supplementary Equation 1 and 2, we have:

$$l(R^2 - \pi r^2) = \frac{nM_{Pd}}{\rho_{Pd}}$$

$$R^2 = \frac{M_{Pd}}{l\rho_{Pd}}\mathrm{n} + \pi r^2$$

$$R = \sqrt{\frac{M_{Pd}}{l\rho_{Pd}}n + \pi r^2} \qquad \text{(Supplementary Equation 3)}$$

Given that $\frac{M_{Pd}}{l\rho_{Pd}}$ and πr^2 are constant, the square of MCN diameter increase linearly with Pd precursor amount, in other words, the diameter of MCN increases linearly with the square root of Pd precursor amount.

- **Synthesis of coaxial-like Au-Pd MCNs with jagged shell**

Coaxial-like Au-Pd MCNs with jagged shell were synthesized by using Na_2PdCl_4 as the Pd precursor as follows. Briefly, in a 20 ml vial, a 1 ml aqueous solution of Au nanorod chains was mixed with a 1 ml of 0.2 M CTAB aqueous solution and then diluted with 7.4 ml of water. Then a 0.4 ml of 10 mM Na_2PdCl_4 aqueous solution was added, followed by adding a 0.2 ml of 0.1 M AA aqueous solution. After gentle shaking, the vial was capped and left in a 30 °C water bath for 10 hours. The samples after growth were washed twice with water at 7000 rpm for 15 mins before characterization and further use.

- **Synthesis of coaxial-like Au-Pd MCNs with truncated octahedron shell**

Coaxial-like Au-Pd MCNs with truncated octahedron shell were synthesized as follows. In a 20 ml vial, a 1 ml aqueous solution of Au nanorod chains was mixed with a 1 ml of 0.2 M CTAB aqueous solution and then diluted with 5.5 ml of water. Then a 1.6 ml of 10 mM H_2PdCl_4 aqueous solution was added, followed by adding a 0.8 ml of 0.1 M AA aqueous solution and a 0.1 ml of 0.1 M HCl aqueous solution. After gentle shaking, the vial was capped and left in a 30 °C water bath for 10 hours. The samples after growth were washed twice with water at 7000 rpm for 15 mins before characterization and further use.

- **Synthesis of coaxial-like Au-Pd MCNs with porous shell**

Coaxial-like Au-Pd MCNs with porous shell were synthesized with the same procedure used for preparation of coaxial-like Au-Pd MCNs with cuboid shell, except for that 0.1 ml of 0.2 M CTAC aqueous solution was used as surfactant instead of CTAB for the synthesis of coaxial-like Au-Pd MCNs with porous shell with all shell thicknesses.

- **Synthesis of coaxial-like Au-Pt MCNs**

Coaxial-like Au-Pd MCNs with different shell thicknesses were synthesized as follows. In 5 different 20 ml vials, a 1 ml aqueous solution of Au nanorod chains was mixed with a 1 ml of 0.2 M CTAB aqueous solution, and diluted with 7.7 ml, 7.4 ml, 6.8 ml, 5.6 ml and 3.2 ml water, respectively. Then 0.1ml, 0.2 ml, 0.4 ml, 0.8 ml and 1.6 ml of 10 mM K_2PtCl_4 aqueous solution were added, followed by adding 0.2 ml, 0.4 ml, 0.8 ml, 1.6 ml and 3.2 ml of 0.1 M AA aqueous solution. After gentle shaking, the vials were capped and left in a 30 °C water bath for 10 hours. The samples after growth were washed twice with water at 7000 rpm for 15 mins before characterization and further use.

- **Synthesis of coaxial-like Au-Ag MCNs**

Coaxial-like Au-Ag MCNs were synthesized with the same procedure used for preparation of coaxial-like Au-Pt MCNs with cuboid shell, except for that 0.1ml, 0.2 ml, 0.4 ml, 0.8 ml and 1.6 ml of 10 mM $AgNO_3$ aqueous solution were used as precursors for the synthesis of coaxial-like Au-Ag MCNs with different shell thicknesses.

- **Synthesis of coaxial-like Au-Pt/Ni MCNs**

Coaxial-like Au-Pt/Ni MCNs with various shell compositions were synthesized with a hydrazine reducing method[3] described as follows. Briefly, in 5 different 20 ml vials, a 1 ml aqueous solution of the Au nanorod chains was mixed with a 1 ml of 0.2 M CTAB aqueous solution, followed by adding different amount of precursors. The specific amount of precursor aqueous solution used for preparing samples in Supplementary Fig 20a-e were listed as follows: (a) 0.3 ml of $NiCl_2$, (b) 0.1 ml of K_2PtCl_4 and 0.1 ml of $NiCl_2$, (c) 0.1ml of K_2PtCl_4 ml and 0.3 of ml $NiCl_2$, (d) 0.1 ml of

K_2PtCl_4 and 0.6 ml of $NiCl_2$, (e) 0.3 ml of K_2PtCl_4 and 0.1 ml of $NiCl_2$. The solution were then diluted with 7.55 ml, 7.7 ml, 7.4 ml, 6.95 ml and 7.4 ml of water respectively. After 0.15 ml, 0.1 ml, 0.2 ml, 0.35 ml and 0.2 ml of 1 M fresh prepared hydrazine aqueous solution were added, the vials were gently shaken and then kept undisturbed at a 50 °C water bath for 10 hours. The samples after growth were washed twice with water at 7000 rpm for 15 mins before characterization and further use.

- **Synthesis of coaxial-like Au-Cu_2O MCNs**

Coaxial-like Au-Cu_2O MCNs were synthesized with a modified hydrazine reducing method described as follows. In two separate vials, a 1 ml aqueous solution of Au nanorod chains was mixed with a 9 ml of 1 wt % PVP aqueous solution and incubated for 1 hour. For different Cu_2O shell thicknesses, a 0.15 ml and a 0.3 ml of 10 mM $Cu(NO_3)_2$ aqueous solution were added respectively. The vials were placed in an ice bath with vigorous magnetic stirring. Then a 0.224 ml of 1 M NaOH aqueous solution and a 0.2 ml of 0.55 M hydrazine aqueous solution were sequentially added. The solutions were kept stirring for 10 mins, and then centrifuged at 5000 rpm for 15 minutes. The precipitates were redispersed in water for characterization and further use.

- **Synthesis of coaxial-like Au-CeO_2 MCNs**

Coaxial-like Au-CeO_2 MCNs were synthesized with an EDTA mediated CeO_2 coating method[7] described as follows. An EDTA-NH_3 aqueous solution was prepared by adding a 0.38 ml of ammonium hydroxide aqueous solution (30 wt %) in 40 ml of water, followed by the dissolution of 0.4 mmol EDTA. For CeO_2 coating with different shell thicknesses, 0.2 ml, 0.175 ml, 0.15 ml and 0.1 ml aqueous solution of Au nanorod chains were added into 4 different 20 ml vials. Then a 8 ml of 25 mM CTAB aqueous solution, a 1.4 ml of EDTA-NH_3 aqueous solution and a 0.14 ml of 10 mM $Ce(NO_3)_3$ solution were sequentially added to each vial. After gentle shaking, the vials were kept in an oven at 90 °C for 5 hours. The samples after growth were centrifuged at 5000 rpm for 15 mins and redispersed in water for characterization and further use.

- **Synthesis of core-shell type Au-CeO_2 MCNs**

Core-shell type Au-CeO_2 MCNs were synthesized with the same procedure used for preparation of coaxial-like Au-CeO_2 MCNs. Briefly, a 200 ml solution of as-synthesized Au nanorods were concentrated to 10 ml and transferred to a 250 ml glass bottle. Then a 160 ml of 25 mM CTAB aqueous solution, a 28 ml of EDTA-NH_3 aqueous solution and a 2.8 ml of 10 mM $Ce(NO_3)_3$ solution were sequentially added to each vial. After gentle shaking, the vials were kept in an oven at 90 °C for 5 hours. The samples after growth were centrifuged at 5000 rpm for 15 mins and redispersed in water for characterization and further use.

- **Synthesis of CeO_2 nanoparticles**

Pure CeO_2 nanoparticles were synthesized with the same procedure used for preparation of coaxial-like Au-CeO_2 MCNs except for that 10 ml water was first added instead of 10 ml solution of Au nanorods.

- **Dissociation of chains of coaxial-like MCNs**

To dissociate the chains of coaxial-like MCNs and collect individual MCNs, the samples after metal/metal oxide growth were centrifuged and washed with THF twice at 6000 rpm for 15 mins to thoroughly remove water, and then redispersed in DMF for characterization and further use.

- **Preparation of PEO-tethered Au nanorods**

Preparation of PEO modified Au nanorods with different PEO graft density were performed as follows: 50 ml aqueous solution of as-synthesized Au nanorods was concentrated to 20 ml by centrifugation and mixed with 0.5 ml, 1 ml, 2 ml and 5 ml aqueous solution of 5 mg ml^{-1} PEO respectively. After gentle stirring for 12 hours, these solutions were centrifuged and washed with water twice at 9000 rpm for 15 mins to remove free PEO, and then redispersed in 10 ml water for further use. They were denoted as 0.5-PEO-Au, 1-PEO-Au, 2-PEO-Au and 5-PEO-Au respectively.

- **Pd deposition on PEO-tethered Au nanorods**

Pd deposition on PEO-tethered Au nanorods with different PEO graft-density were performed as follows. In 4 different 20 ml vials, 1 ml of 0.5-PEO-Au, 1-PEO-Au, 2-PEO-Au and 5-PEO-Au were mixed with a 1 ml of 0.2 M CTAB aqueous solution and 7.55 ml of water respectively. Then a 0.3 ml of 10 mM H_2PdCl_4 aqueous solution was added, followed by adding a 0.15 ml of 0.1 M AA aqueous solution to each of the vials. After gentle shaking, the vials were capped and left in a 30 °C water bath for 10 hours. The samples after growth were washed twice with water at 7000 rpm for 15 mins before characterization and further use.

Core-shell type Au@Pd MCNs with thick shell were synthesized using the same approach as described above except for that a 0.8 ml of 10 mM H_2PdCl_4 aqueous solution was used as precursors in all the synthesis.

- **Preparation of Au nanosphere chains**

Au nanosphere chains were prepared as follows. Typically, a 50 ml aqueous solution of as-synthesized Au nanospheres was concentrated to 0.3 ml through centrifugation and then added drop-wise into a 10 ml solution of thiol-terminated polystyrene in THF with a concentration of 1 mg ml^{-1}. The solution was sonicated for half an hour and incubated overnight. After 8 cycles of washing with THF, the modified Au nanospheres were redispersed in a 5 ml of DMF in a 20 ml vial. A 2.4 ml solution of DMF/water (40% wt/60% wt) was then added drop-wise under gentle shaking and the solution was held at room temperature overnight. The mixture solution was dialyzed against 800 ml of 50 mM CTAB aqueous solution using dialysis membrane tubing (molecular-weight-cutoff of 6000−8000 g mol^{-1}) for 2 hours to further stabilize the chains, followed by centrifugation at 7000 rpm for 15 mins. The precipitates were redispersed in 7.4 ml of water. The aqueous solution of Au nanosphere chains was used for all secondary growth.

- **Synthesis of Saturn-like Au-Pd MCNs**

Saturn-like Au-Pd MCNs with different shell thicknesses were synthesized as follows. Briefly, in 3 different 20 ml vials, a 1 ml aqueous solution of Au nanosphere chains was mixed with a 1 ml of 0.2 M CTAB aqueous solution, and then diluted with 7.85 ml, 7.7 ml and 7.4 ml of water

respectively. Then 0.1 ml, 0.2 ml and 0.4 ml of 10 mM H_2PdCl_4 aqueous solution were added, followed by adding 0.05 ml, 0.1 ml and 0.2 ml of 0.1 M AA aqueous solution. After gentle shaking, the vials were capped and left in a 30 °C water bath for 10 hours. The samples after growth were washed twice with water at 8000 rpm for 15 mins before characterization and further use.

To dissociate the chains and collect isolated Saturn-like Au-Pd MCNs, the samples after growth were washed with THF twice at 8000 rpm for 15 mins, and redispersed in DMF for characterization and further use.

- **Synthesis of Saturn-like Au-Pt MCNs**

Saturn-like Au-Pt MCNs with different shell thicknesses were synthesized as follows. Briefly, in 3 different 20 ml vials, a 1 ml aqueous solution of Au nanosphere chains was mixed with a 1 ml of 0.2 M CTAB aqueous solution, and then diluted with 7.7 ml, 7.4 ml and 6.8 ml of water respectively. Then 0.1 ml, 0.2 ml and 0.4 ml of 10 mM K_2PtCl_4 aqueous solution were added, followed by adding 0.2 ml, 0.4 ml and 0.8 ml of 0.1 M AA aqueous solution. After gentle shaking, the vials were capped and left in a 30 °C water bath for 10 hours. The samples after growth were washed twice with water at 8000 rpm for 15 mins before characterization and further use.

- **Synthesis of Saturn-like Au-Ag MCNs**

Saturn-like Au-Ag MCNs were synthesized with the same procedure used for preparation of Saturn-like Au-Pt MCNs, except for that 0.1ml, 0.2 ml and 0.4 ml of 10 mM $AgNO_3$ aqueous solution were used as precursors for the synthesis of Saturn-like Au-Ag MCNs with different shell thicknesses.

- **Synthesis of core-shell type Au-Pt MCNs**

Core-shell type Au-Pt MCNs were synthesized with the same procedure used for preparation of coaxial-like Au-Pt MCNs, except for that 1 ml of Au nanorod solution (concentrated from 5 ml as-synthesized solution of Au nanorods) were used as seeds instead of 1 ml of Au nanorod chains solution.

- **Photocatalytic degradation of MB**

The polymers on coaxial-like Au-Pt MCNs were first removed using the same sonication and washing method as described in the main text and then dissolved in water. For the photocatalytic degradation of MB, 0.2 ml of catalyst suspension (containing 0.058 mg Au, determined by inductively coupled plasma optical emission spectrometry) was added to a solution containing 0.25 ml of 0.1 M $NaCO_3$, 1 ml of methanol, 3.75 ml of water and 0.2 ml of 0.6 mM MB. For the photocatalytic degradation, the solution was irradiated with visible light (300 W xenon lamp, λ>420 nm) without stirring. Cooling water was used to eliminate the thermal effect in the reaction. The rate of MB degradation was evaluated using UV−vis spectroscopy (PERKIN LAMBDA 40 UV-Vis system).

Supplementary References

1. Jiang, R., Qin, F., Ruan, Q., Wang, J. & Jin, C. Ultrasensitive Plasmonic Response of Bimetallic Au/Pd Nanostructures to Hydrogen. *Adv. Funct. Mater.* **24**, 7328-7337 (2014).
2. Sánchez-Iglesias, A. et al. Rapid Epitaxial Growth of Ag on Au Nanoparticles: From Au Nanorods to Core–Shell Au@Ag Octahedrons. *Chem. Eur. J.* **16**, 5558-5563 (2010).
3. Grzelczak, M., Rodríguez-González, B., Pérez-Juste, J. & Liz-Marzán, L.M. Quasi-Epitaxial Growth of Ni Nanoshells on Au Nanorods. *Adv. Mater.* **19**, 2262-2266 (2007).
4. Liu & Guyot-Sionnest, P. Synthesis and Optical Characterization of Au/Ag Core/Shell Nanorods. *J. Phys. Chem. B* **108**, 5882-5888 (2004).
5. Nikoobakht, B. & El-Sayed, M.A. Preparation and Growth Mechanism of Gold Nanorods (NRs) Using Seed-Mediated Growth Method. *Chem. Mater.* **15**, 1957-1962 (2003).
6. Jana, N.R., Gearheart, L. & Murphy, C.J. Seeding Growth for Size Control of 5−40 nm Diameter Gold Nanoparticles. *Langmuir* **17**, 6782-6786 (2001).
7. Li, B. et al. (Gold Core)@(Ceria Shell) Nanostructures for Plasmon-Enhanced Catalytic Reactions under Visible Light. *ACS Nano* **8**, 8152-8162 (2014).
